# Implications of short time scale dynamics on long time processes

**DOI:** 10.1063/1.4996448

**Published:** 2017-12-22

**Authors:** Krystel El Hage, Sebastian Brickel, Sylvain Hermelin, Geoffrey Gaulier, Cédric Schmidt, Luigi Bonacina, Siri C. van Keulen, Swarnendu Bhattacharyya, Majed Chergui, Peter Hamm, Ursula Rothlisberger, Jean-Pierre Wolf, Markus Meuwly

**Affiliations:** 1 Department of Chemistry, University of Basel, Klingelbergstrasse 80, 4056 Basel, Switzerland; 2 Department of Applied Physics (GAP), University of Geneva, 22 Ch. de Pinchat, 1211 Geneva 4, Switzerland; 3 Institute of Chemical Sciences and Engineering, EPFL, Lausanne, Switzerland; 4 Department of Chemistry, University of Zurich, Zurich, Switzerland

## Abstract

This review provides a comprehensive overview of the structural dynamics in topical gas- and condensed-phase systems on multiple length and time scales. Starting from vibrationally induced dissociation of small molecules in the gas phase, the question of vibrational and internal energy redistribution through conformational dynamics is further developed by considering coupled electron/proton transfer in a model peptide over many orders of magnitude. The influence of the surrounding solvent is probed for electron transfer to the solvent in hydrated I^−^. Next, the dynamics of a modified PDZ domain over many time scales is analyzed following activation of a photoswitch. The hydration dynamics around halogenated amino acid side chains and their structural dynamics in proteins are relevant for iodinated TyrB26 insulin. Binding of nitric oxide to myoglobin is a process for which experimental and computational analyses have converged to a common view which connects rebinding time scales and the underlying dynamics. Finally, rhodopsin is a paradigmatic system for multiple length- and time-scale processes for which experimental and computational methods provide valuable insights into the functional dynamics. The systems discussed here highlight that for a comprehensive understanding of how structure, flexibility, energetics, and dynamics contribute to functional dynamics, experimental studies in multiple wavelength regions and computational studies including quantum, classical, and more coarse grained levels are required.

## I. INTRODUCTION

Many fundamental processes in chemistry, biology, and physiology occur on time scales slower than microseconds, but they have their origin in dynamics on the femto- to picosecond time scale. An example is the generic time scale of a chemical reaction in solution which occurs on a typical time scale of a second. However, the actual elementary process (bond formation/bond breaking) is a femto- to picosecond process. The ultimate reasons for this large span of time scales (12–15 orders of magnitude) are diffusion and the inefficient coupling of translational and thermal motion of the atoms (primarily translational degrees of freedom) to the coordinate(s) along which the reaction progresses.

The purpose of this review is to highlight topical examples in which information and insight from short-time dynamics have implications for processes occurring on considerably longer time scales. One such example is enzyme catalysis.^1^ For adenylate kinase (AdK), fluctuations on the picosecond time scale have been linked with dynamics on the micro- to millisecond. Experimentally, thermoAdk (a hyperthermophilic homologue) and mesoAdk (a mesophilic homologue) show different turnover rates at the same temperature. The comparative analysis of the dynamics between the two types of enzymes at atomic resolution enabled identification of atomic fluctuations that are crucial for the activity. NMR relaxation experiments were used to connect the dynamics on the picosecond to those on the millisecond time scale. This allowed us to connect different tiers^2^ of the energy landscape. The correspondence of ps to ns flexibility in the hinges between mesophilic and hyperthermophilic Adk at temperatures at which enzymatic activities are matched provided the link between local, fast-timescale dynamics, and slower global dynamics.

Triggering events such as illumination by light provide energy, which can be supplied to a system of interest in various ways and in different degrees of freedom (dof). The most natural origin is thermal energy which, however, is often unspecific and inefficient to evoke a particular response. Other possibilities include photoexcitation (flash photolysis,^3^ electronic excitation), pH-change, collisional excitation, or the excitation of molecular vibrations.^4^ As a consequence of such a change, processes including catalysis or further protein-ligand binding can take place due to redistribution of the available energy. Understanding the link between the cause and effect and following the system's dynamics as a function of time are of fundamental importance for fully characterizing the function of a complex system and move toward molecular design.

In situations where multiple elementary steps take place between the initial preparation and the finally observable state, the sequence of events and the nature, structure, and stability of potential intermediates may also be unknown. For example, for the homodimeric hemoglobin HbI from *Scapharca inaequivalvis*, experiments^5–11^ and atomistic simulations^12–14^ have found that the functionally relevant dynamics between the deoxy (tense, T, ligand unbound) and the oxy (relaxed, R, ligand bound) state is strongly influenced by structural changes at the interface and the number of water molecules between the two monomers. The most significant tertiary structural change concerns the orientation of the phenyl-sidechain of Phe97 in which the *χ* angle changes from 50° to 160° upon ligand binding. The conformational change is accompanied by a change in the degree of hydration of the interface, in that 17 water molecules are present in the deoxy state and only 11 water molecules are found in the ligand-bound protein. One open question is whether side-chain rotation drives water diffusion or *vice versa*.

For another physiologically relevant process, sensing environmental signals, and rapid metabolic response in bacteria, it is known that a “two-component” signal transduction systems^15^ consist of a histidine protein kinase that transfers a phosphoryl group to a conserved aspartate of a response regulator (RR) protein, which modulates its activity. The diguanylate cyclase PleD of *Caulobacter crescentus* is such a response regulator.^16–19^ The protein synthesizes the bacterial second messenger cyclic di-guanylic monophosphate (c-di-GMP),^18^ a molecule of great interest, which regulates surface-adhesion properties and motility in bacteria.^20^ In order to carry out its function, several elementary steps (protein dimerization, activation through phosphorylation, and allosteric (auto)inhibition) need to take place.^21^ However, the sequence in which these elementary steps occur is unknown but of fundamental importance to understand the mode of action of this protein.

The reasons for the wide spanning time scales between cause and outcome are the (inefficient) coupling between degrees of freedom to transfer energy between them (often the dofs into which energy can be injected differ from those which are relevant for the ensuing functional dynamics), the (slow) conformational dynamics which is a “search problem” on high-dimensional, the rough potential energy surfaces (PES),^22^ or energy dissipation due to friction. Such processes lead to a marked slowdown in transmitting the primary information into a productive channel. Hence, one of the distinguishing properties of an efficient system is the fact that the energy input is converted into a productive functional motion/transformation and not simply dissipated as heat into the system and its environment. For processes in solution, a further complication concerns entropic effects, which can lead to additional slowdown (“entropy traps”).^23,24^ However, contrary to enthalpy, which can be directly influenced by tuning and exploiting intermolecular interactions, the direct control of entropy is difficult. Finally, it is also possible that the “initial” and “final” states are connected through multiple pathways as in the photocycle of bacteriorhodopsin^25,26^ or in ligand rebinding to neuroglobin.^27^ This leads to a further loss of flux between the initial state and the desired final state due to multiple pathways and potential branching of the routes between them.

**FIG. 1.**
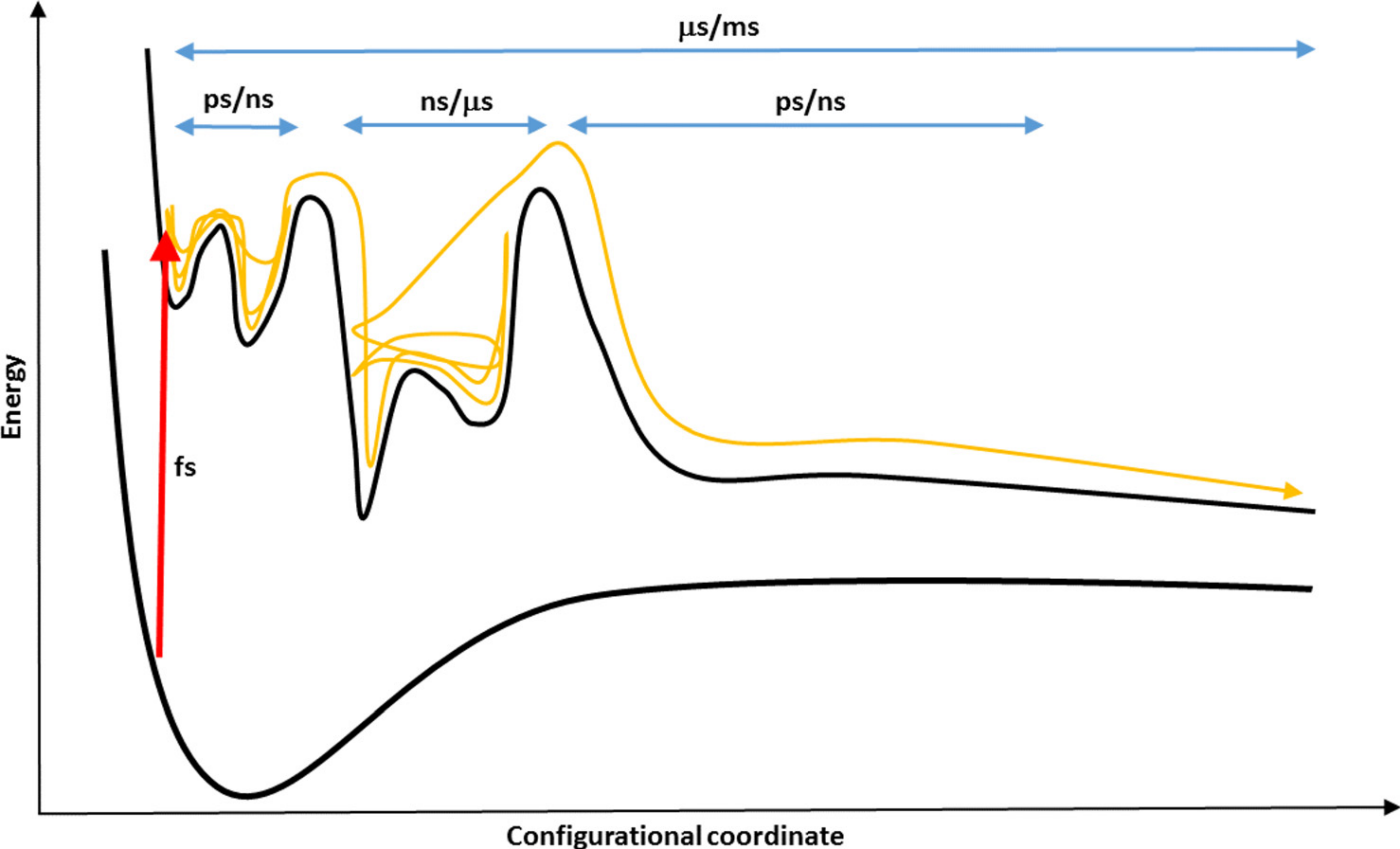
Illustration of a photoinduced process (red arrow) and the ensuing conformational dynamics on the excited state potential energy surface spanning several orders of magnitude. The orange curve tracks the excited state dynamics in different basins and on different time scales.

The present review focuses on the processes occurring on multiple time scales in complex systems.^28^ As will become clear, starting from a system in thermodynamic equilibrium and providing energy into a particular channel will only lead to the desired output if the energy transport along the particular pathways can be controlled. This is usually achieved by understanding the available energy migration pathways, how efficient they are in reaching the desired final state, and whether the mapping between the initial and final state is unique or not. While the energy exchange between particular modes is relatively well studied,^29–33^ the transport of “information” is less well studied. Some notable efforts concerned the characterization of the causality between the correlated motions by information theoretical means^34^ or changes in the protein conformational entropy upon ligand binding.^35^ Hence, the concepts of structure, coupling, energy migration, and entropy on given time scales are intimately linked, and a multidisciplinary approach is required to arrive at a refined molecular picture and understanding. As will also become clear, achieving this can be tremendously difficult. However, understanding, controlling, and eventually guiding energy flow and information exchange between two given end points of a pathway are required to solve this problem (Fig. 1).

Sections II–IV will discuss individual systems of increasing complexity and size and on increasingly long time scales. They include single molecules and peptides in the gas phase and range up to small proteins in solution to large proteins in cellular contexts.

## II. SYSTEMS DYNAMICS FROM FEMTOSECONDS TO MILLISECONDS

### A. Small gas phase systems: Vibrationally induced photodissociation

Decomposition products from energized molecules in the atmosphere are of great practical relevance. In the troposphere, interaction of stable and reactive species with sunlight leads to a myriad of products, which are involved in a rich chemistry. Of particular importance are sulfur-containing species such as H_2_SO_4_ and their decomposition products, which lead to long-living, harmful (re)agents, which affect the ozone layer and which are involved in acid rain and aerosol formation in the tropo- and stratosphere.^36^ Because H_2_SO_4_ cannot decay along a thermal or electronically driven pathway in the atmosphere, alternative reaction mechanisms are needed to be considered. It was suggested^37^ and later confirmed^38–41^ that a vibrationally induced mechanism can drive the H_2_SO_4_ → SO_3_ + H_2_O reaction. In addition, vibrationally induced reactivity has also been investigated in mode-selective chemistry.^4,42^

Vibrational overtone induced reactions differ in their reaction kinetics from thermally driven reactions. Hence, the way in which energy is provided to a system (random thermal vs. directed) can determine the fate of the energized molecule. The population of an electronically excited state leads to reaction kinetics that can be analyzed within a Rice-Ramsperger-Kassel-Marcus (RRKM) framework. This differs from the population of excited vibrational states which leads to a non-equilibrium preparation of the system from which it usually decays in a non-statistical, non-RRKM fashion.^29^ In the atmosphere, vibrationally excited molecules can undergo unimolecular reactions before vibrational quenching occurs due to collisions with the surrounding material.^43–45^ Especially, X-H stretching modes (X = O, C, N) are of particular interest.^43,45^ A well-studied example is the dissociation of HNO*_x_.*^43,46,47^ Vibrationally induced reactivity leads to improved atmospheric models.^43^ One example is the formation of atmospheric aerosols, which influences the climate, due to hydrophilic acids and alcohols in the atmosphere, whereas for models in which vibrational overtone-induced photodissociation is excluded, incorrect particle sizes are obtained.^45^

One topical example for vibrationally induced reactivity is the photodissociation of pyruvic acid.^48,49^ For this system, it was demonstrated that if the decomposition reaction is initiated through pumping of the OH-stretch vibration, the kinetics does not follow an RRKM scheme unlike the thermally induced reaction. This is insofar important, as the chemistry following a thermally or vibrationally driven process differs. For pyruvic acid, the thermal process generates methylhydroxy-carbene which subsequently can isomerize to acetaldehyde. Contrary to that, the vibrational overtone excitation produces relatively stable high-energy methylhydroxy-carbene radicals, which can drive further reactions through collisions with other collision partners, such as water.^50^

A computational investigation of the H_2_SO_4_ → SO_3_ + H_2_O reaction was undertaken by utilising multisurface adiabatic reactive molecular dynamics (MS-ARMD).^40,51^ This surface crossing algorithm combines fitted empirical force fields with energy-dependent weighting functions to obtain a global reactive potential energy surface (PES) capable of treating several competing reaction pathways. This is necessary in the investigation of H_2_SO_4_, since the fragmentation pathway competes with an intramolecular H-transfer reaction of similar barrier height. From several thousand independent (*NVE*) trajectories with a maximum simulation time of 1 ns, it was found that the fast OH overtone vibration (period T≤10 fs) induces elimination on the pico- to nanosecond time scale, depending on the level of excitation of the OH-stretch. The final state analysis of the trajectories results in an energy distribution that can assist in the identification of the products.

**FIG. 2.**
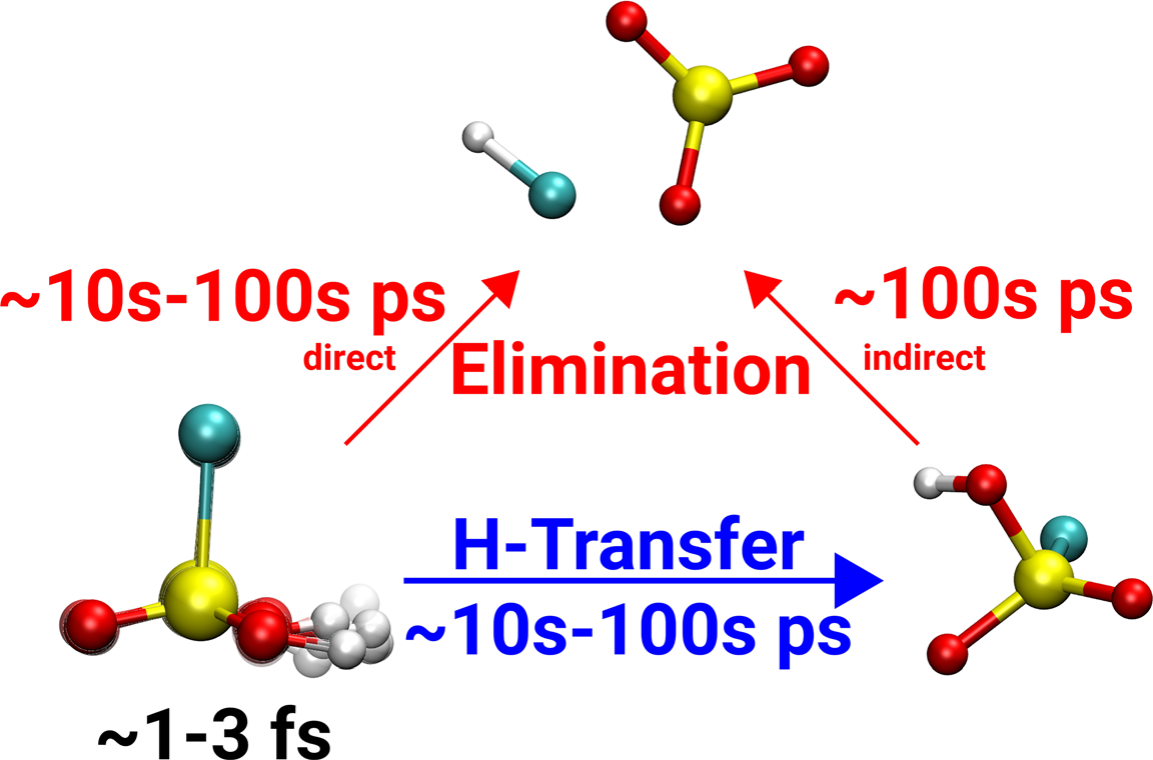
Schematic representation of the possible reaction paths of HSO_3_Cl, including approximate time scales for a high excitation energy ($\nu_{\text{OH}}=5$ and 6).

Since direct experimental verification for OH-stretching overtone induced photofragmentation of H_2_SO_4_ is difficult in the laboratory due to practical reasons (e.g., ready deprotonation of H_2_SO_4_ in the presence of water), derivatives of sulfuric acid have been considered as a proxy for establishing such a vibrationally induced reaction mechanism. Therefore, MS-ARMD simulations were also carried out for chloro- and fluorosulfonic acid (HSO_3_Cl and HSO_3_F) (Fig. 2).^52,53^

When comparing the reaction dynamics in HSO_3_F and HSO_3_Cl, it was found that even for moderate excitation levels ($\nu_{\text{OH}}=4$) along the OH-stretch coordinate, HCl elimination in HSO_3_Cl occurs for 15% of the trajectories on a 2.5 ns time scale.^52^ This compares with no elimination at all for HSO_3_F for the same level of excitation and over the same time interval after vibrational excitation. The computed MP2/6 − 311++G(2d,2p) barriers for HCl and HF elimination and H-transfer for the two systems are 35.1 kcal/mol vs. 33.1 kcal/mol and 38.1 vs. 38.1 kcal/mol for HSO_3_F and HSO_3_Cl, respectively.^52^ Hence, the barrier height for HF or HCl elimination, respectively, cannot be the reason why HF elimination does not occur in HSO_3_F. For a better understanding, statistical simulations within the RRKM (Rice-Ramsperger-Kassel-Marcus) framework were carried out for both molecules. A collision free regime was assumed, and tunneling was neglected. For the energy range equivalent to an excitation with ν=4 to 6, it was found that the RRKM rates for HSO_3_F are only a factor of 5 smaller compared with the rate for HSO_3_Cl. Such a small difference is in marked contrast with the results from explicit MD simulations, and suggests that the origin for the different behaviors is not predominantly statistical but rather dynamical. By comparing with the H_2_O elimination dynamics from H_2_SO_4_, it is thus found that HSO_3_Cl is a suitable proxy, whereas HSO_3_F is not.

The reactive dynamics following vibrational overtone excitation of small gas phase systems leads to productive (photodissociation) and unproductive events (converting the energy into motion). Next, a system is considered, which is prepared in a “locked” (or frustrated) conformational state and which requires dynamics on very long time scales compared with the elementary step of interest to find a state suitable for the reaction to proceed.

### B. Medium sized gas phase system: Dynamics in HG_3_W observed over 8 orders of magnitude in time

Proton-coupled electron transfer (PCET) reactions are important and ubiquitous in biology, as they enable energy conversion and storage in photosynthesis^54^ and in the respiratory chain,^55^ mediate charge transfer (CT),^56^ and are involved in DNA repair.^56^ However, direct studies extending from the time scale for electron transfer until the point when the proton transfer is completed have been scarce. A recent example for such an investigation used a silver-containing metal-peptide cation synthetized from histidine (H), glycine (G), and tryptophan (W): [HG3W + Ag]^+^. For this system, it was possible to observe the overall PCET dynamics spanning 8 orders of magnitude in time.^57^ The experiment was based on the combination of mass spectroscopy and pump-probe optical spectroscopy. After electrospray ionization, the sample is trapped for 200 ms in a high-pressure (5 mTorr) chamber inside the mass spectrometer. To cover the extremely long time span of the process investigated, femtosecond lasers with optical delay lines and nanosecond lasers with electronic time-synchronization are employed.

**FIG. 3.**
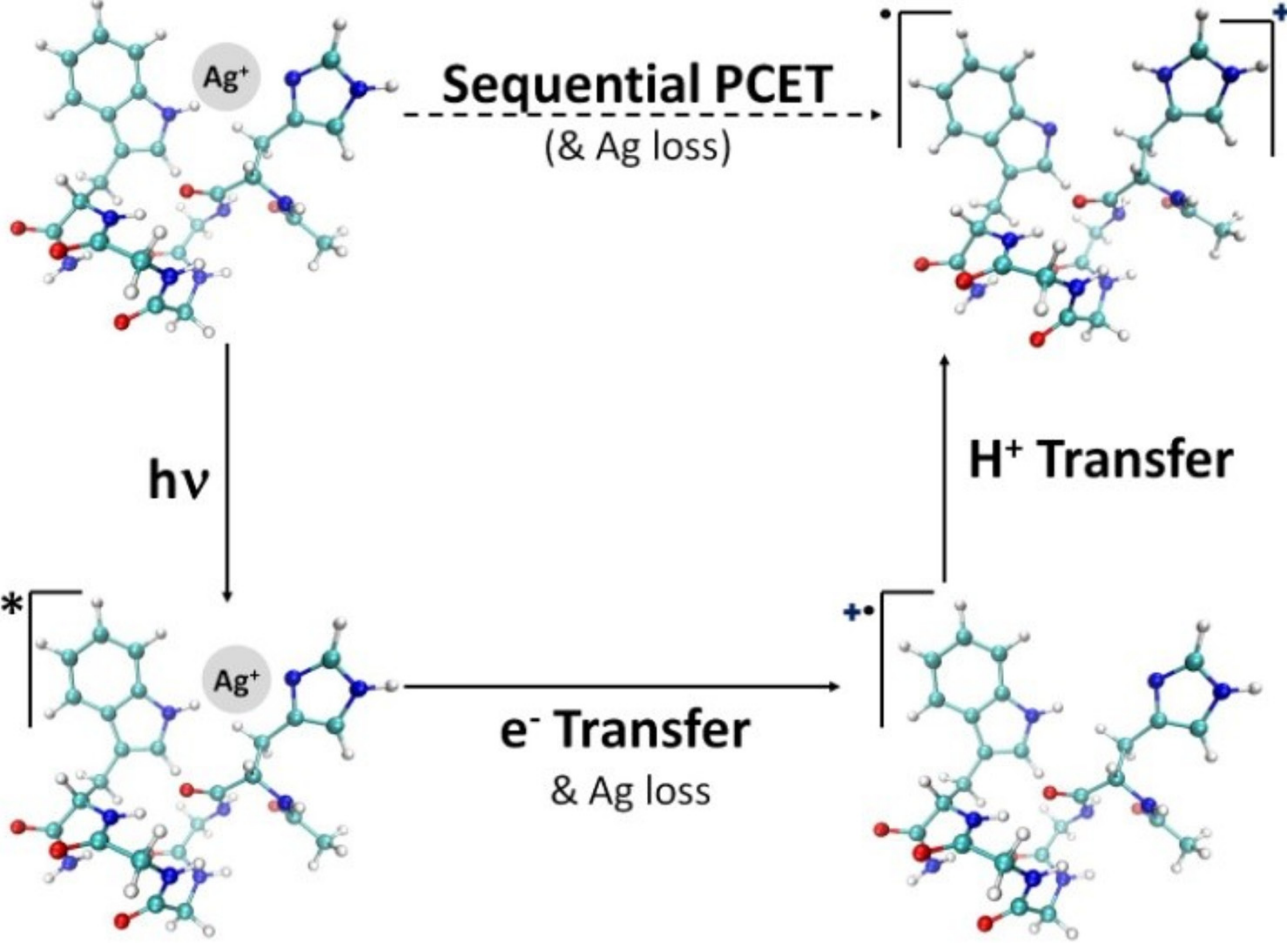
Schematic representation of PCET dynamics in [HG_3_W + Ag]^+^ metalpeptide complexes. Irradiation at 266 nm initiates an electron transfer from tryptophan to Ag^+^, leading to the loss of Ag. Electron transfer is followed by proton transfer from tryptophan to histidine with formation of a distonic ion. The peptide structures are schemes and do not correspond to calculated structures. Reproduced with permission from MacAleese *et al.*, J. Am. Chem. Soc. **138**, 4401–4407 (2016). Copyright 2016 American Chemical Society.

By analyzing the time-resolved traces of (i) silver-containing against silver-free ions over the first 30 ps and (ii) the evolution of the fragmentation yield of the radical peptide [HG3W]$\cdot^{+}$ up to 1 ms, it was possible to monitor electron and proton transfer on their respective time scales. The final picture which emerges is the following: initially, a $\pi \to \pi^{*}$ transition of tryptophan is excited upon 266 nm photo-excitation. This in turn leads to electron transfer from the peptide to the silver cation to form Ag^0^. Now the charge and radical are localized on tryptophan (Fig. 3). After this short initial dynamics (3.5 ps), a proton is transferred from tryptophan to histidine on a time-scale of the order of hundreds of microseconds.

**FIG. 4.**
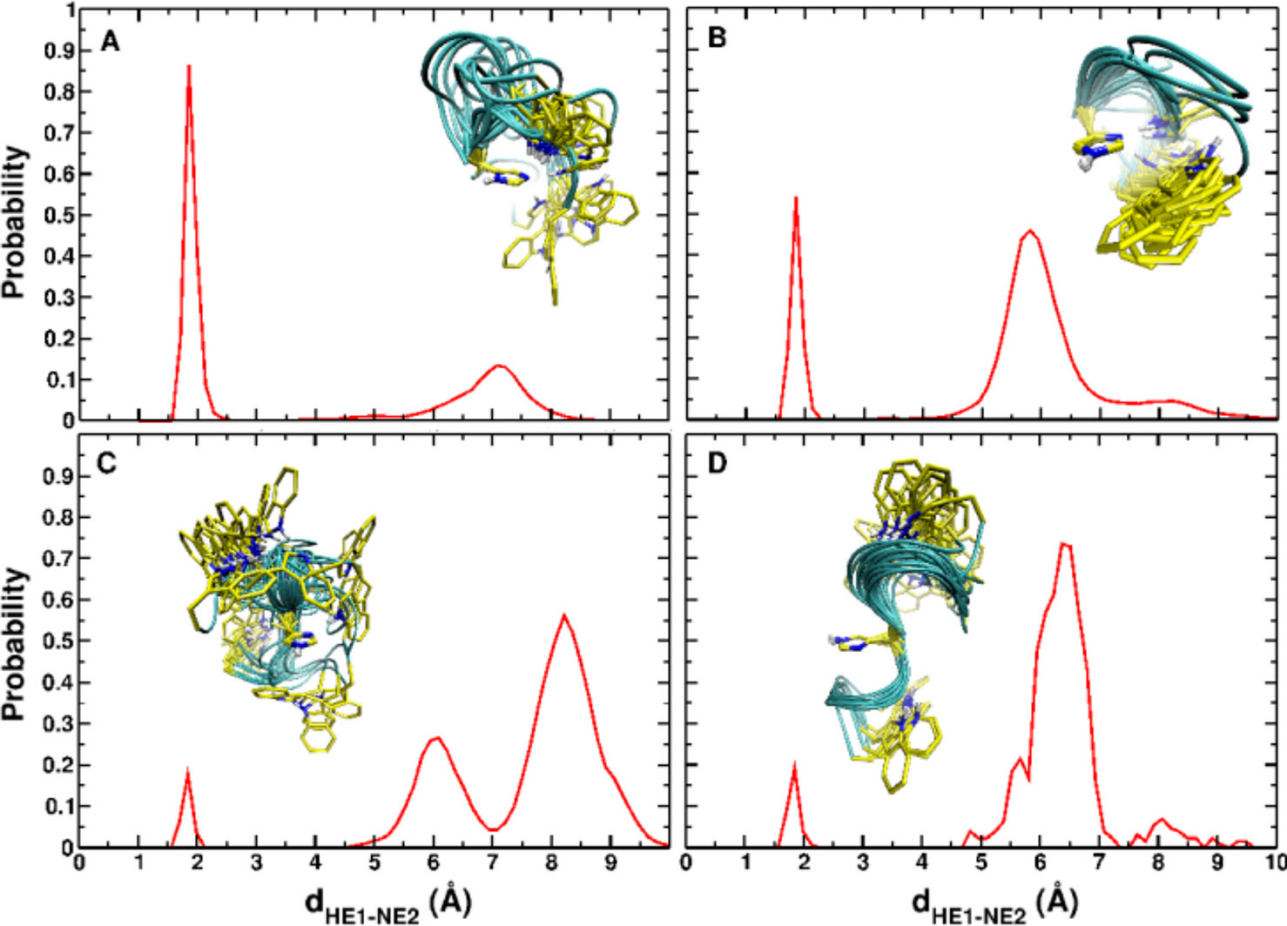
Indole_NH_–imidazole_N_ distance distribution in HG_3_W^.+^ over 50 independent runs of 10 ns each, starting from an extended structure (A) and two compact structures (B and C) obtained from the [HG_3_W + Ag]^+^ complex. (D) Same but over 8 independent runs of 1 *μ*s each, starting from structures in C. Insets show superpositions of structures from MD trajectories. Reproduced with permission from MacAleese *et al.*, J. Am. Chem. Soc. **138**, 4401–4407 (2016). Copyright 2016 American Chemical Society.

Because the rate limiting step (conformational dynamics or proton transfer) cannot be determined directly from experiment, molecular dynamics simulations and electronic structure calculations were carried out. Although the rate of formation of a proton-transfer reactive structure can be as short as a few nanoseconds for such a structure, this time-scale is strongly influenced by the initial peptide conformation. In particular, the presence of Ag^+^ in [HG3W + Ag]^+^ entails a series of structural changes including an unfavorable orientation of the tryptophan side chain with respect to histidine and the presence of several H-bonds, which lock the initial conformation, hindering the formation of a proton-transfer-competent structure (Fig. 4). Indeed, the calculations based on this conformation do not predict the occurrence of proton-transfer up to 8 *μ*s, consistent with a major slowing-down of the proton-transfer process as observed experimentally. Hence, conformational dynamics and not the proton transfer barrier height is the rate limiting step in the overall process.

Up to this point, gas-phase systems were considered, including a solvent environment that increases the number of available degrees of freedom considerably and leads to additional relaxation channels. In order to disentangle intra- from intermolecular dynamics in solution, it is important to describe the dynamics of the solvation shell first. This is discussed in the next example which deals with atomic ions in water. The use of atomic solutes excludes any internal degrees of freedom.

### C. Solvent dynamics following charge transfer

In a chemical reaction in solution, the solvent molecules are not spectators but respond to the change of electronic structure of the reactants by minimizing the free energy. During this process, the solvent molecules rearrange around the solute in what has been named as solvation dynamics.^58^ One of the most extreme cases of solvation dynamics are charge transfer reactions since the oxidation state change of the reactants induces dramatic changes in the field of forces with the solvent species.

The first step of intermolecular charge transfer (CT) reaction (e.g., electron or proton transfer) between a donor and an acceptor in solution is a charge transfer to the solvent. Charge-transfer-to-solvent (CTTS) states, which are quasi-bound states of the solute-solvent system with no equivalent for the isolated ions, are ideal objects to investigate this first step. The CTTS states are commonly found for aqueous halides and, since the latter lack internal (nuclear) degrees of freedom, the CTTS dynamics is entirely governed by the structure and motion of the solvent species. The CTTS states are therefore ideal probes of the solvation dynamics upon an electronic excitation of a solute. The process has been investigated by ultrafast Transient Absorption and photoemission (PE) spectroscopies, which probed the early time dynamics of aqueous CTTS states.^59–61^ These studies were in part obscured by the strong signal of the solvated electron, which has hindered the observation of subsequent dynamics. Recently,^62–64^ the Chergui group introduced two new types of observables of the CTTS: ultrafast fluorescence of the CTTS states and ultrafast X-ray absorption spectroscopy (XAS) at the core transitions of the halide.

**FIG. 5.**
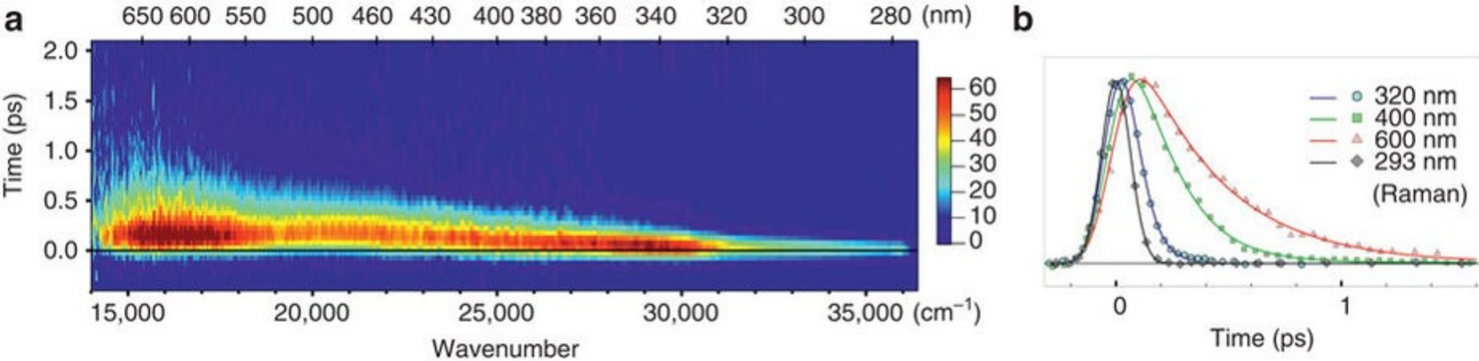
Femtosecond fluorescence of aqueous iodine. (a) Fluorescence of 1 M NaI dissolved in water upon 266 nm excitation. The Raman signal from water was removed from the plot. (b) Normalized kinetic traces at different wavelengths with their representative fits (continuous lines), compared with the Raman signal from water at 293 nm, whose temporal width gives the IRF of the setup. Reproduced with permission from Messina *et al.*, Nat. Commun. **4**, 2119 (2013). Copyright 2013 Macmillan Publishers Ltd.

Ultrafast fluorescence would provide a direct measurement of the electron departure to the solvent since photons are emitted during the time the ground and the excited (CTTS) state wave functions still overlap. The time-wavelength plot^62^ of the CTTS fluorescence for aqueous I^−^ is shown in Fig. 5. An emission spanning from the UV (approximately 300 nm) to the visible region (approximately 670 nm) appears promptly at time zero. Its decay is strongly wavelength-dependent going from ca. 60 fs at λ<330 nm to ∼400 fs at 650 nm. These results reveal the very large inhomogeneity of the excited centres with each iodide having a somewhat different solvent shell at *t* = 0. The starting solvent configuration will determine the subsequent dynamics where the solvent accommodates the excited species, giving rise to very different Stokes shifts spanning over 1 eV. The redder the emission, the larger is the Stokes shift and, therefore, the more stable the configuration, which is reflected in a longer decay time.

**FIG. 6.**
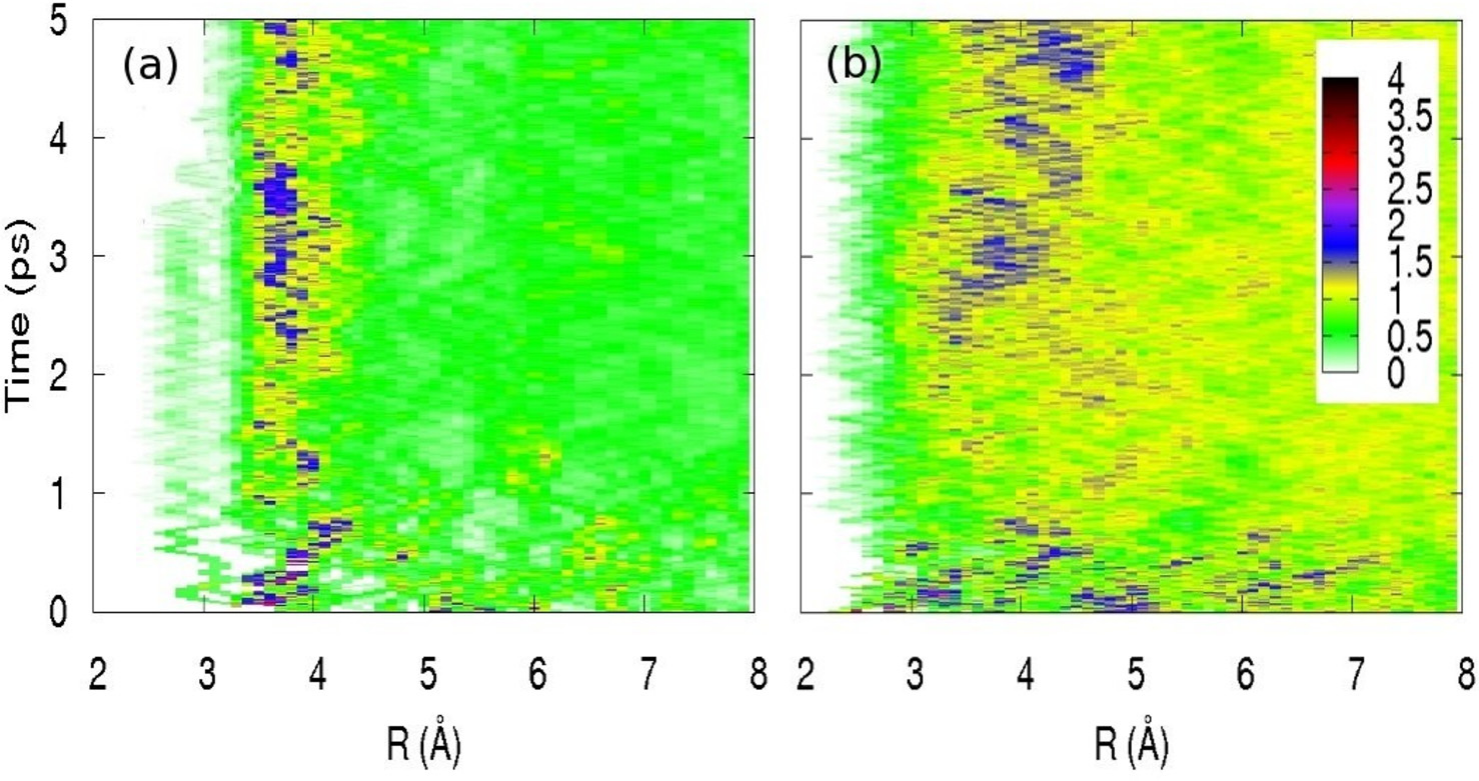
(a) Time evolution of the I^0^–O radial distribution for the first 5 ps following the removal of an electron from iodide. These distributions are obtained from an average over 15 starting configurations over the entire time range; (b) same for I^0^–H. Reproduced with permission from Pham *et al.*, J. Am. Chem. Soc. **133**, 12740–12748 (2011). Copyright 2011 American Chemical Society.

The ejected electron leaves a neutral iodine behind whose interaction with the water molecules is totally different from that of the parent iodide. Indeed, the latter is known to have a hydrophilic solvation, while neutral iodine has a hydrophobic interaction. The solvation dynamics should lead to dramatic solvation shell rearrangements. The picosecond L1 and L2 edge absorption studies indeed confirmed a significant solvation shell change,^63,64^ while the femtosecond ones confirmed a prompt departure of the electron.^64^ Full quantum, hybrid quantum-classical and full classical simulations of the solvation dynamics were used to reproduce the picosecond XAS data with very good agreement. They also allow to retrieve the evolution of the solvation shell from a hydrophilic to a hydrophobic one, which is well captured by the simulations of the evolution of the I–O and I–H radial distribution functions (RDFs) as a function of time shown in Fig. 6. The ejection of the electron at *t* = 0 leads to a very significant change in the solvation structure of the I–H RDF, which requires about 2–3 ps to recover some order. On the other hand, the I–O RDF shows an expansion of the I–O distances on average, while one molecule forms an I–OH_2_ loosely bound complex that lives for ca. 3 ps. The complex then dissociates and the water molecule merges with the solvation shell. The simulations show that the solvation shell around neutral iodine is formed when the statistics of H-bonds reach a plateau, in a clear manifestation of hydrophobicity. Indeed, the latter is not characterised by a driving force between solute and solvent, but by entropy, i.e., the affinity of water molecules to form H-bonds and to exclude the solute.

For a single ion in solution, electronic degrees of freedom are the only internal variables (I^0^ vs. I^−^). Hence, the next level of complexity arises for systems with a large number of internal and external degrees of freedom for which additional couplings and energy exchange pathways become available. Two different scenarios are considered: first, a monomeric protein (PDZ2) in solution in which the dynamics can be initiated through an external perturbation; second, a chemical modification in dimeric insulin is discussed, which necessitates a meaningful parametrization of the intermolecular interactions. This is done from short time simulations (hundreds of picoseconds) for the parametrization of the force field with respect to experimental data, and then the nanosecond dynamics of the dimeric protein is considered and compared with the experiment.

## III. SMALL SOLVATED PROTEINS: PDZ DOMAIN AND INSULIN

### A. Local perturbations to mimic allosteric dynamics

Allostery is the coupling between two spatially separated binding sites of a protein and is an important mechanism that Nature uses to regulate the affinity of certain substrates to a protein, thereby controlling the metabolism. According to the conventional view of allostery, a conformational change of the protein (that might however be very small^65^) is the source of a signal, but it should be noted that other mechanisms have been proposed as well, which are based exclusively on the dynamical properties.^66^ In any case, the binding of a ligand at a so-called *allosteric site* increases (or decreases) the affinity for a substrate at a distant *active site*. Hence, an allosteric protein can be viewed as a “transistor,” and complicated feedback networks of many such switches ultimately make up a living cell.^67^

**FIG. 7.**
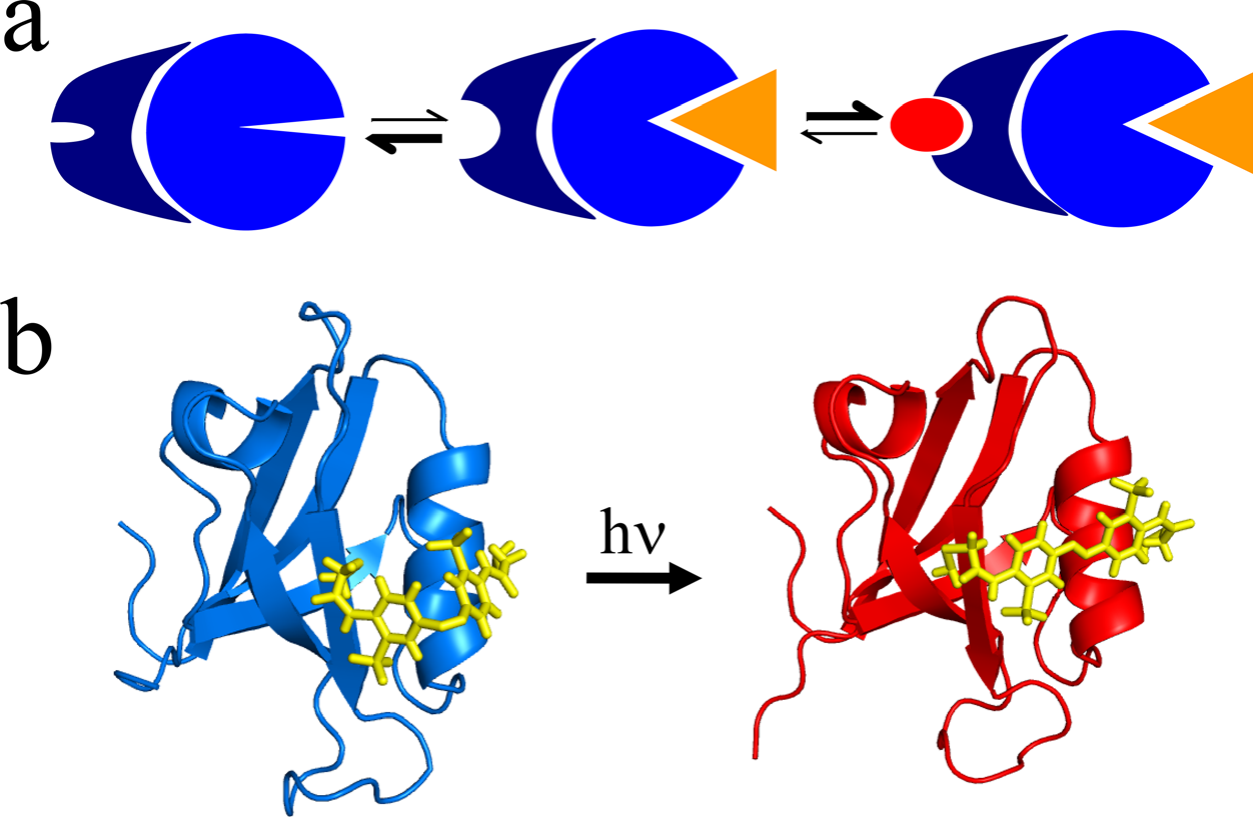
(a) The “textbook” explanation of allosteric regulation: A ligand (orange) binds to the allosteric site of a protein, thereby changing the binding affinity for a substrate at a distant active site (red). (b) A PDZ2 domain with an azobenzene-moiety linked across the ligand binding groove (PDB entries 2M0Z and 2M10).

The textbook explanation of allostery is depicted in Fig. 7(a). From the point of view of allosteric regulation, this figure might appear sufficient, i.e., what we need to know is if the change in binding affinity depends on whether or not a ligand is bound to the allosteric site. This is essentially the level of description of the Monod-Wyman-Changeux (MWC) model to explain cooperative binding of oxygen to hemoglobin—the prototype example of allosteric regulation.^10^ However, from a microscopic, atomistic point of view, Fig. 7(a) does not give a clear picture on how, why, and how fast the transition occurred. Given the complexity of the regulation network in a living cell, the switching speed, albeit probably being fast on the biological timescale, might not be irrelevant.

The equivalent of the level of description of Fig. 7(a) applied to the protein folding problem would reduce the latter to a folding free energy. Again, from a biological perspective, this is often sufficient, as all we need to know is how stable a protein is and what its folded structure is. Nevertheless, the protein folding problem has, of course, been tackled on a much more microscopic level, leading to concepts such as the folding funnel, rugged energy landscapes, down-hill folders, and folding networks.^68–73^ The emerging view of allostery works on the hypothesis that any protein exists as a conformational ensemble, and that a conformational change upon ligand binding is the result of a shift in populations within that ensemble.^74,75^

With the one exception of hemoglobin, the dynamics of the structural transition giving rise to allosteric regulation has not been investigated with high time resolution. Despite the fact that it is not its natural function, hemoglobin is photo-switchable in its natural form through the photo-dissociation of a heme ligand, enabling experiments with a time resolution reaching the 10 s of femtoseconds. It has been found that tertiary conformational changes occur in the time range from 1 ns to 1 *μ*s in a highly non-exponential manner, whereas quaternary changes are slower.^10^

Hamm and co-workers recently set out to develop tools to initiate an allosteric response in a protein that is not *per se* photoswitchable,^76–78^ applying a concept that has been introduced first by Woolley and co-workers.^79–81^ To that end, an azobenzene-derivative is used as a photoswitch, which can be isomerized on a very fast 1 ps timescale between its *cis* and a *trans* conformer with light of different wavelengths. The switch is chemically designed such that it can be covalently linked to virtually any position at the protein surface via two cysteines. Hence, upon photo-isomerization, one may apply a force between the two points of a protein in an extremely well-controlled manner. In the past, the concept has mostly been used to initiate folding/unfolding of small peptides^82,83^ or proteins,^84^ and we now set out to apply it to a bi-stable system, i.e., switching between the two states of an allosteric protein.

Hamm and co-workers chose the second PDZ (PDZ2) domain from human tyrosine-phosphatase 1E (hPTP1E) for these studies, which has been demonstrated to possess allosteric properties,^85^ and which has served as a model system for allostery for a long time. Possible signal transduction pathways and mechanisms have been widely investigated by MD simulations as well as bioinformatics approaches.^86–95^ The PDZ2 domain is a small 96 residue protein with a binding groove between the *α*_2_-helix and the *β*_2_-strand [see Fig. 7(b)]. Binding of a ligand results in a small but measurable structural change with an RMSD of the binding groove of ≈0.4 Å, according to X-ray crystallography.^96^

**FIG. 8.**
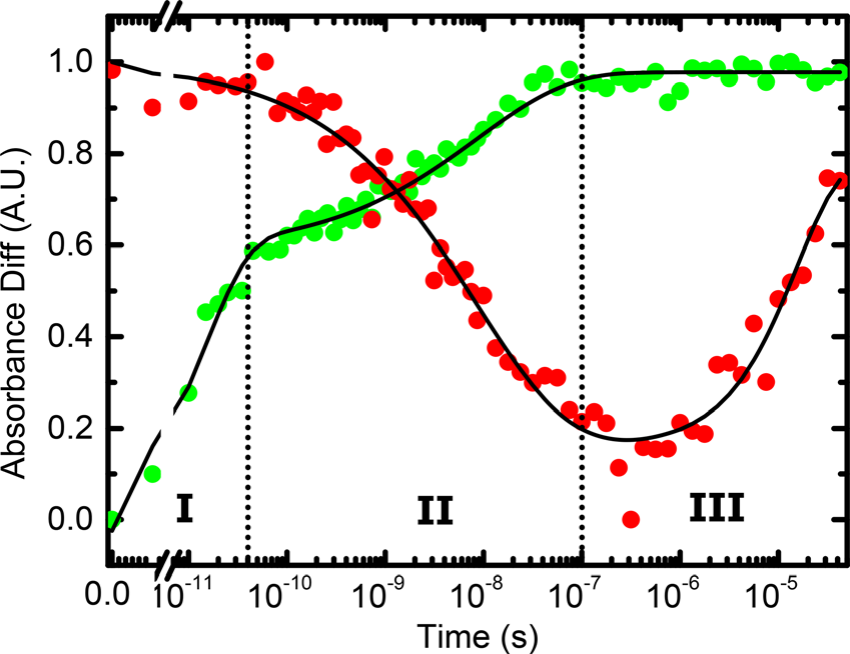
Response of the PDZ2 domain after perturbing the binding groove with the help of an azobenzene-crosslinker, measured by transient IR spectroscopy. The red data show the response of the protein as a whole via the amide I band, and the green data that of a vibrational mode localized on the azobenzene-crosslinker. Adapted with permission from Buchli *et al.*, Proc. Natl. Acad. Sci. U. S. A. **110**, 11725–11730 (2013). Copyright 2013 National Academy of Sciences, U.S.A.

Transient IR spectroscopy has been used to study the response of the protein upon photo-triggering the azobenzene-crosslinker (Fig. 8).^76^ The initial event, i.e., the photo-isomerization of the azobenzene-moiety, is a barrierless photochemical reaction that occurs on a 1 ps timescale.^97^ It triggers a cascade of events in the protein, which covers orders of magnitudes in time. Three phases can be distinguished: During phase I, up to ≈40 ps, the heat deposited into the protein as a result of the photo-isomerization of the cross-linker, which dissipates about 3 eV of vibrational energy, cools into the solvent. During phase II, from ≈40 ps to ≈100 ns, the binding groove opens, observed via the response of a vibrational mode localized on the azobenzene-crosslinker (Fig. 8, green). Finally, during phase III, beyond ≈100 ns, more remote parts of the protein adapt to the perturbation, seen as a delayed response of the amide I band, which reports on the structure of the protein backbone (Fig. 8, red).

**FIG. 9.**
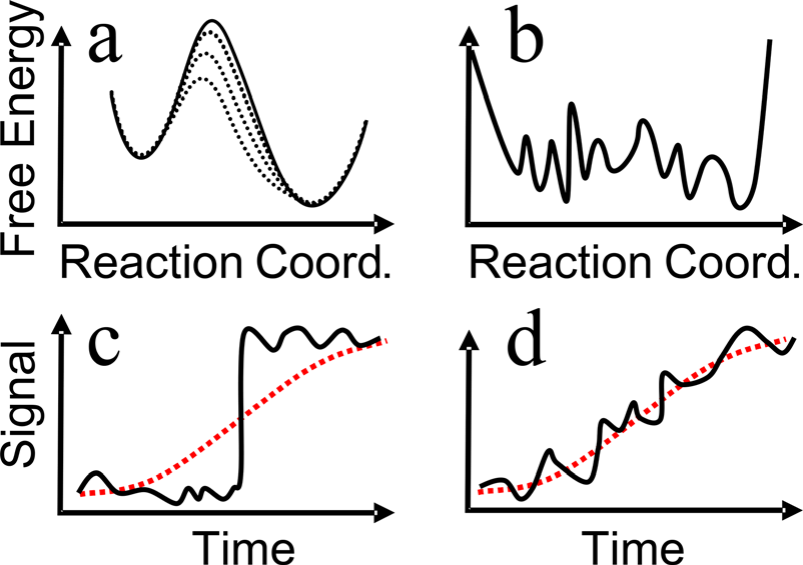
Mechanism of the allosteric response. The two mechanisms are discussed in the text.

In Refs. 76 and 77, Hamm and co-workers have mostly focused on phase II, i.e., the opening of the binding groove, which occurs in a highly non-exponential manner and by itself covers 3.5 orders of magnitudes in time. Non-exponential protein dynamics have been discussed extensively, for instance, in the context of ligand (CO) dissociation and rebinding in hemoglobin or myoglobin.^98,99^ Two limiting scenarios are typically discussed: A parallel process is characterized by a distribution of exponential decay processes, originating from a distribution of barrier heights in an inhomogeneous ensemble of proteins [Fig. 9(a)]. In this case, individual single-molecule trajectories would still behave as a two-state system with either a closed or an open binding groove, and one would observe essentially sudden jumps between these two states [Fig. 9(c), black]. The distribution of jump times would be non-exponential, revealing a non-exponential response after ensemble averaging [Fig. 9(c), red]. In the opposite limit, the system diffuses on a rugged, high dimensional energy landscape [Fig. 9(b)], which commonly leads to non-exponential response as well.^100^ In this case, single-molecule trajectories would essentially be equivalent with the average, apart from statistical noise, without large jumps [Fig. 9(c), black versus red]. This is indeed what has been observed in accompanying MD simulations.^76^ It was furthermore found that water friction is in part the source of the ruggedness of the protein energy landscape.^76,77^ This result agrees with the view that the allosteric response is related to a shift in populations between the substates of an ensemble of protein conformations.^74,75^ The response thus shares many properties with downhill folding.^70,101,102^ The response of the azobenzene-crosslinked PDZ2 domain was also studied computationally, using molecular dynamics (MD) simulations and transition networks, revealing a semi-quantitative agreement with experiment.^28,78,103^ The atomistic details of the MD simulations open the possibility to elucidate the structural dynamics of the process.

Figure 8 also illustrates a limitation of this work. While it was possible by chance to isolate one vibrational mode localized on the photo-switch (Fig. 8, green), which enabled us to draw very specific conclusions on the binding groove dynamics,^76,77^ the response of the amide I band (Fig. 8, red) averages over the whole protein without any site-specific information. While MD simulations^78,103^ suggest that these slower processes are related to conformational changes in the floppy parts of the protein (i.e., termini and loop regions), and as such might actually be related to the allosteric signalling in “remote” parts of the protein, direct experimental support for such an interpretation is still lacking. Currently, work concerns the incorporation of non-natural amino-acids containing isolated vibrational labels, similar to Refs. 104 and 105, which eventually should allow to retrieve site-selective information from transient IR spectroscopy in a versatile manner.

### B. Halogenated insulin: Functional studies based on accurate models from short time scale simulations

Linking time scales in a different context is afforded by a discussion of recent work related to the dynamics and energetics of halogenated insulins. Halogenation has been found to modify the aggregation and binding behaviour of insulin under physiological conditions. In order to lay out a roadmap for further modification and optimization of the hormone, a molecular-level understanding of the energetics and dynamics of insulin in various aggregation states is required. The system considered here is an iodinated insulin in which a hydrogen atom on the ring of TyrB26 was replaced by an iodine atom.^106^ Such modified insulins have been found to exhibit an improved binding of insulin monomer toward its receptor.^107,108^ For a molecular-level understanding of the insulin dimer disassembly dynamics (into two monomer) and the binding of the monomer toward the receptor (or a model thereof), atomistic simulations are a meaningful complement to experimental characterization. This was attempted for iodo-TyrB26-modified insulin.

**FIG. 10.**
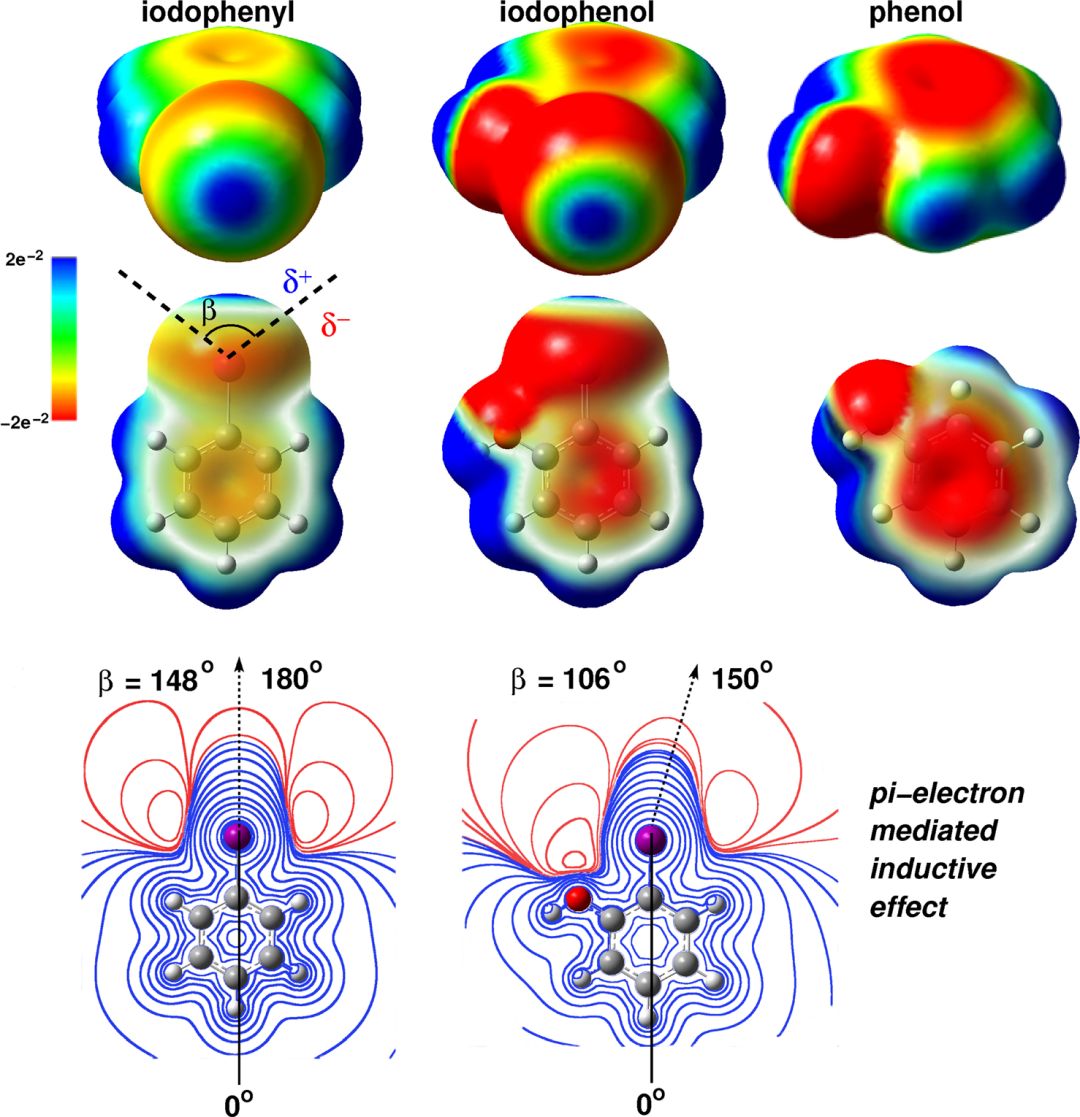
Top: Electrostatic-potential (ESP) surface maps of phenol, iodophenol, and iodophenyl at the 0.001e Bohr^−3^ isodensity. The color scale of the ESP ranges from −2.12 (10^−2^) (red) through 0 (green) to 2.12 (10^−2^) (blue). In the upper row, the iodine (facing the viewer) exhibits the effect of the electron-donating OH on the *σ*-hole. The lower row shows the effects of iodine on the π− system of the phenol ring. The angle *β* represents the σ− hole size as delimited by black dashed lines. $\delta^{+}$ and $\delta^{-}$ represent the respective regions of positive and negative charge around the iodine. Bottom: ESP contours of iodophenyl (left) and 2-iodophenol (right), at different isovalues, calculated in the plane of the aromatic ring. The black dashed arrow indicates directionality of the C-I bond.

Mixed quantum mechanical/molecular mechanics (QM/MM) simulations would, in principle, be the method of choice for such a problem. However, given the size of the system, the high level of electronic structure calculations required, and the extended time scales on which the system needs to be followed, such an approach is currently not viable. Instead, the problem was decomposed into several parts. First, for the chemically relevant fragment (iodo-phenyl, see Fig. 10), a molecular mechanics representation was determined consistent with experimental observables. Then, this model was embedded in the hormone, and MD simulations for the monomer, dimer, and the complex with the insulin-*μ* receptor were carried out.

For this, the model compounds were first parametrized from *ab initio* calculations and fitted to available experimental data which included hydration free energies $\Delta G_{\text{hyd}}$, heat of vaporization, and the pure solvent density. Accurate hydration free energies can be obtained from simulations of the pico- and nanosecond dynamics around the solute.^109^ The electrostatic multipole model (MTP)^110^ was obtained for the phenolic ring of TyrB26 and iodophenolic ring of I-TyrB26. Atomic multipoles are assigned to all heavy atoms (but not the hydrogens). The parametrization protocol followed a recently developed strategy which includes optimization of multipole moments to best represent the electrostatic potential and van der Waals parameters to correctly describe the experimental solution phase data, including the hydration free energy of iodophenol.^111,112^ A parametrization based on such data can be expected to describe the most relevant interaction modes between solute and solvent in a meaningful way to allow quantitative simulations for the situation in the protein.

**FIG. 11.**
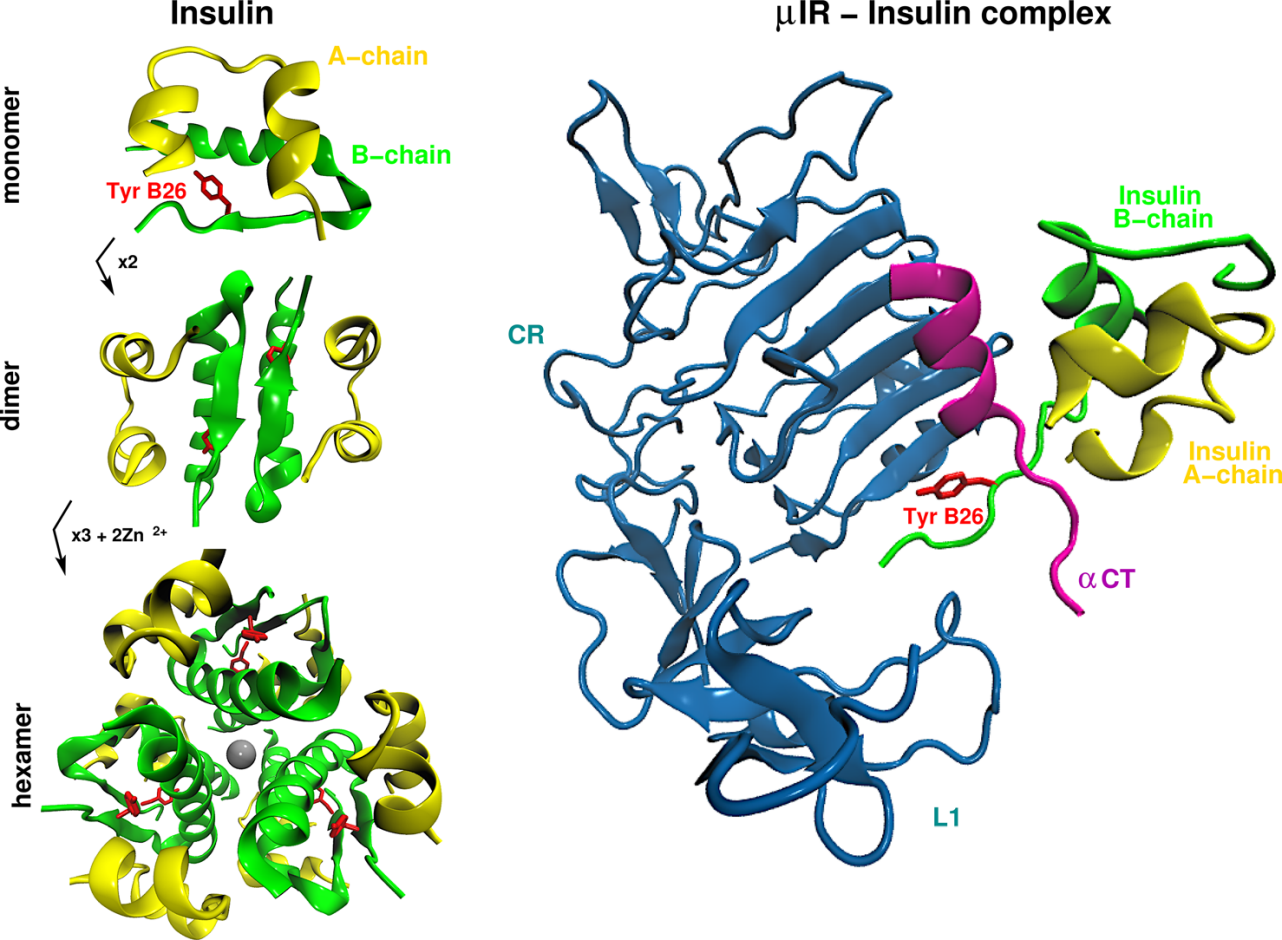
Structure of Insulin and receptor domain. Left panels: The monomeric hormone (A and B chains, top panel) forms zinc-free dimers via anti-parallel association of B chain *α*-helices and C-terminal *β*-strands (brown, middle panel); two zinc ions then mediate assembly of three dimers to form classical hexamer (bottom panel). Right panel: *μ*IR domain (blue) complexed with insulin monomer. The interaction takes place via residue TyrB26 (red) located on the B-chain (green).

In order to guide the experiments, atomistic simulations of insulin dimer and insulin monomer complexed to the insulin-microreceptor (*μ*IR) were carried out. These studies indicated that replacement of one hydrogen atom by an iodine at the ortho position of TyrB26 leads to structural rearrangements at the dimerization interface and enhanced binding to the receptor due to several favourable interactions. These findings were subsequently confirmed by X-ray crystallography and affinity measurements.^106^ In particular, the simulations predicted insertion of the large iodine atom (atomic radius twice larger than that of carbon) within an internal cleft between the A- and B-chains (see Fig. 11). Such accommodation requires a specific reorientation of the B26 side chain. In these simulations, the internal location of the iodine atom within a protomer was found to be compatible with native-like assembly of the dimer interface within the hexamer with a subtle reorganization of successive aromatic-aromatic interactions. These predictions were then verified by determining the crystal structure of a 3-I-TyrB26-insulin hexamer at 2.3 Å resolution (pdb code 5EMS).^106^ The nonpolar packing of the B26 iodo-aromatic ring is thus reminiscent of the packing in the WT system.

The binding mode of 3-I-TyrB26 toward the *μ*IR was also probed based on the structure of the monomer-*μ*IR complex. Remarkably, these simulations predicted the formation of favorable halogen bonding and halogen-directed hydrogen bonding between the modified B26 ring and the receptor. At this point, it is important to mention that accompanying simulations with point charge models only find one favourable contact between iodine and the environment at the receptor interface which highlights the need for improved, multipolar electrostatic models when considering halogenated compounds. Such directional electrostatic interactions, exploiting the *σ*-hole of the halogen (here iodine) and its electronegative equatorial band, have previously been observed in crystal structures of specific complexes between proteins and halogenated ligands.^113^

Until this point, the spectroscopic probes used to follow the dynamics of the protein of interest were covalently linked to the protein. However, it is also possible to explore protein interiors with small molecules following photodissociation. The intent is to cover a larger fraction of the available configuration space. In this case, the coupled dynamics of the protein, the ligand, and the surrounding solvent are probed. This constitutes another layer of complexity.

### C. Solvated medium sized systems: Ligand binding in myoglobin

Experimentally, the focus has been on new methods to probe the electronic and structural dynamics of various biological and chemical samples. In particular, the implementation of the first ultrafast deep-UV 2 D-spectroscopy has been a game changer.^114,115^

As far as proteins are concerned, this method allows to unravel the hitherto unknown electron-transfer (ET) processes from photoexcited tryptophan to the heme in ferric myoglobins (Mb)^116^ and in ferrous ones, such as deoxy-Mb,^117^ while only fluorescence resonant energy transfer (FRET) has been assumed so far. The time scales for ET are relatively slow, about 20–30 ps. These studies were recently extended to ligated Mbs, but in order to avoid the interference of time scales due to ET and to ligand dynamics, Chergui *et al.* used an IR probe. The results vary remarkably with the ligand: with CN, a reduction of the Fe atoms is observed, but some degree of electron density is on the porphyrin; with NO, the electron goes to the latter; while with CO, the electron is predominantly on the porphyrin, but further CO ligand dissociation from the porphyrin anion occurs on a time scale of approximately 200 ps, typical of protein fluctuations. Such a long time scale has been found in a recent picosecond X-ray absorption spectroscopic study, which probed the return of the NO ligand to the Fe atom of the porphyrin.^118^ This process is governed by protein fluctuations.

Computationally, the structural dynamics accompanying NO-rebinding to myoglobin has been recently investigated with the aim to assign the transient, metastable structures relevant for rebinding of the ligand on different time scales. For this, reactive MD simulations using MS-ARMD simulations were run between the bound ^2^*A* and the unbound ^4^*A* state. The energy for each of the states was represented as a reproducing kernel^119–121^ for the subspace of important system coordinates [the heme(Fe)–NO separation and angle, and the doming coordinate of the heme-Fe] combined with an empirical force field for all remaining degrees of freedom.

These simulations yield nonexponential kinetics (see Fig. 9) for ligand rebinding. The time scales (10 and 100 ps) confirm those from optical, infrared experiments, and X-ray absorption and previous computational works.^118,122–132^ The influence of the iron-out-of-plane (Fe-oop or “doming”) coordinate on the rebinding reaction, as predicted by experiment,^125^ was directly established by analyzing the differences in simulations in which the Fe-oop coordinate was treated in an indirect manner and the explicit treatment afforded by the RKHS-interpolated PESs. The two time scales are associated with two structurally different states of the His64 side chain—one “out” (state A) and one “in” (state B)—which control ligand access and rebinding dynamics. Such an unequivocal assignment was not possible from the experiment alone, as the lifetime of these states is too short and their concentration is too low to provide sufficient information for their characterization.^133^ In addition, such simulations provide a molecular explanation why an energetically feasible state for NO-binding to heme is typically not found in Mb: Although the bound Fe-ON state is a local minimum on the potential energy surface, the energy of this state on the unbound ^4^*A* manifold is lower and, hence, the bound ^2^*A* Fe-ON cannot be spectroscopically characterized. Finally, the computed X-ray absorption spectrum (XAS) for the bound and unbound states and the difference between them agree very favourably with the experimentally determined spectrum. However, the experimental spectra are unable to distinguish between structures with photodissociated NO “close to” or “far away” from the heme-Fe in the active site.

In this fashion, validation of experimental results by the MD simulations and in-depth analysis of the configurations driving the dynamics on the different time scales (10 ps and 100 ps) allowed us to identify the structural origins of the conformational dynamics at a molecular level. It is expected that further combined experimental and computational studies of this kind will provide the necessary insight to link energetics, structures, and dynamics in complex systems.

Combining all the above hallmarks of the coupled dynamics in complex systems, Rhodopsin is discussed as a final example. For this system, the initial process—stimulation by light—leads to a physiological response and spans many orders of magnitude in space and time.

## IV. LARGE SYSTEMS: THE VISION DYNAMICS IN LIVE MAMMALIANS

### A. Ultrafast laser-induced isomerization and control of rhodopsin in solution and *in vivo*

**FIG. 12.**
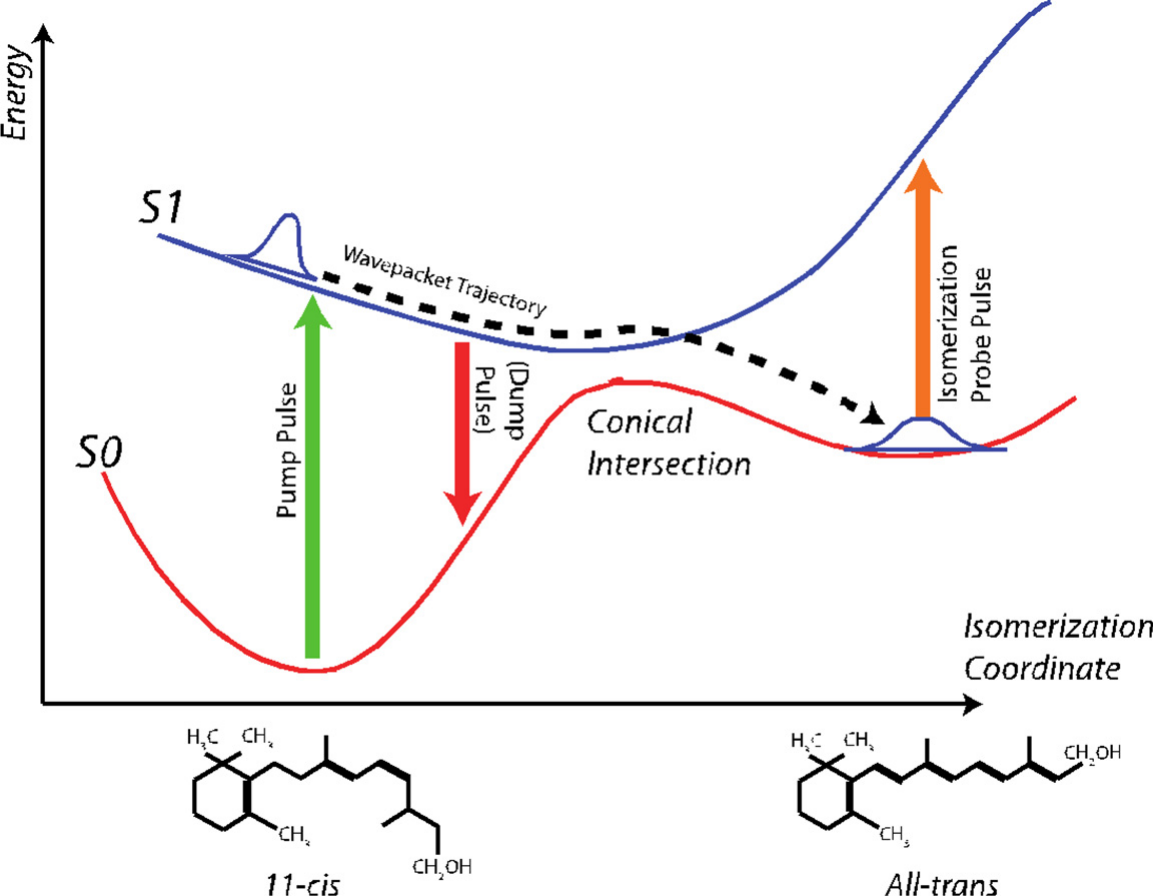
Potential energy surfaces involved in the isomerization of Rhodopsin, going from 11*-cis* to *all trans* upon photon absorption.

The vision process, which entails a long cascade of events going from initial photon absorption to nerve-impulse generation, is triggered by the rhodopsin-bound 11–*cis*-retinal to all-*trans* retinal isomerization (see Fig. 12). The ultrafast investigation of this primary vision step started with the pioneering paper by the Shank group where the arrival in the isomerization state in less than 100 fs was time-resolved using a transient absorption scheme.^134^ Shortly thereafter, Wang *et al.* showed wavepacket oscillations in this molecule, demonstrating that coherence is preserved for at least 2 ps after photo-excitation despite the passage through the conical intersection leading to isomerization.^135^ Gerber's and Cerullo's groups demonstrated that it was possible to transfer the excited population back to the 11–*cis* ground state by stimulated emission before the transition through the conical transition.^136,137^

**FIG. 13.**
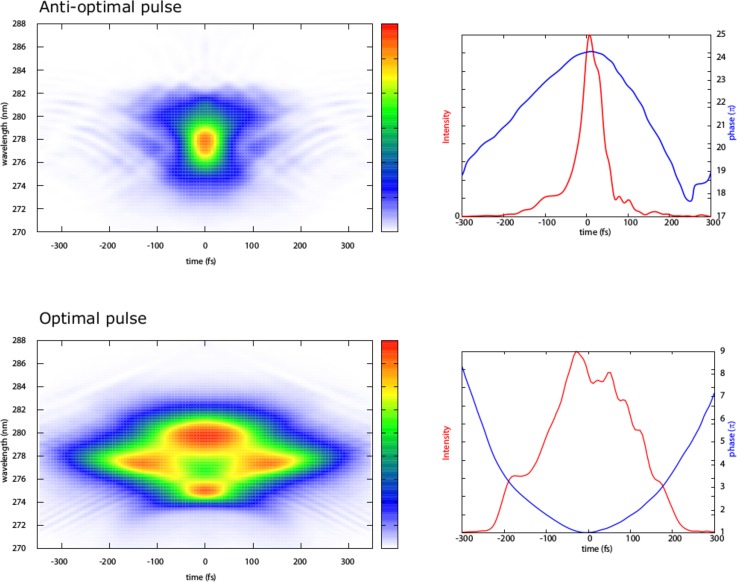
Second Harmonic Generation Frequency Resolved Optical Gating (SHG-FROG) traces associated with the optimal and anti-optimal pulses retrieved by Wolf's group by repeating the original Miller's experiment using phase-only shaping. Note that the experimental method uses second harmonic generation, which results in wavelength being halved. The corresponding temporal intensity profiles and phase functions reconstructed from the experimental FROG traces are also provided.

In 2005, Miller and Prokhorenko provided an experimental demonstration of optimal quantum control of the retinal photo-isomerization in bacteriorhodopsin.^138^ The authors showed that by applying an optimally phase- and amplitude-shaped laser pulse retrieved using a closed-feedback approach, it was possible to increase the retinal photo-isomerization yield by 20% as compared with a Fourier limited femtosecond pulse. Conversely, an anti-optimal pulse shape reduced the photo-isomerization efficiency by 20%. Miller's results demonstrated that the primary vision step could be modulated by the spectral phase of light. Wolf's group repeated this experiment a few years ago. Despite several technical differences (e.g., learning algorithm, pulse shaper technology), this second take, based exclusively on phase-shaping, fully confirmed the findings originally reported. The total modulation efficiency for isomerization is 6%, which is in total agreement with the 5–7% value reported by Miller for phase-only shaping. Interestingly, Wolf's team has observed a significant intensity dependence on the Rhodopsin isomerization yield for a given spectral phase (Fig. 13). Notably, at the lowest fluences, i.e., for the conditions closest to Miller's experiment, the optimal pulse retrieved is >200 fs long and possesses a multi-peak structure as opposed to a short anti-optimal pulse approximately 50 fs long. These pulse shape features are in line with those reported in the original experiment and seem to point, at least for the case of the optimal pulse, to a tailored adaptation of the excitation process to the excited state vibrational manifold of the molecule for controlling its isomerization. However, these works were performed on molecules in solution, while the possibility of extending the phase-sensitive interaction with the primary vision step to the overall vision process in living beings was far from being assessed.

**FIG. 14.**
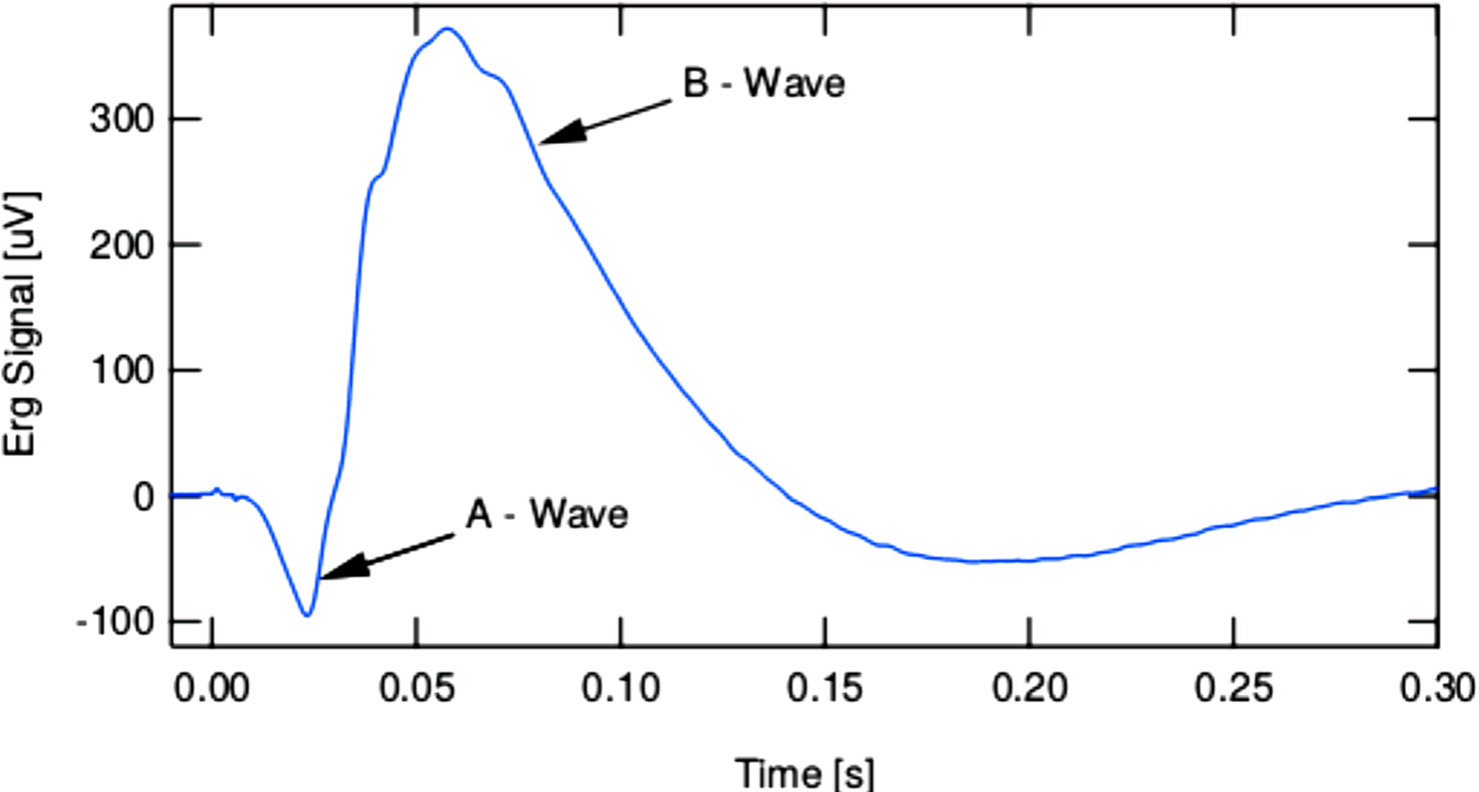
Response of a mouse retina upon excitation by femtosecond pulse obtained by electroretinography. One can identify the a- and b-waves, negative at early times and positive at later times, respectively.

Moving on this pathway, it was recently investigated by Wolf's group that it is possible to manipulate the photo-isomerization yield of photoreceptor molecules in a live mouse by modulating the spectral phase of a green femtosecond light pulse and record the electric signal generated by the retina. Their experiment is based on a kHz amplified Ti:Sapphire laser system coupled with a noncollinear parametric amplifier generating 50 fs pulses at 535 nm. The beam is sent onto the eye of the anesthetized mouse by passing through an index-matching medium to correct for the eye curvature. The experimental read-out relies on electroretinography (ERG). Namely, the visual response is acquired by three electrodes placed in contact with the irradiated eye, the forehead, and under skin in the tail (for ground voltage reference), respectively. The signals are first frequency filtered and pre-amplified. Successively, after being processed by a differential amplifier, the resulting signal is input into a fast oscilloscope and converted into a digital trace. So far, the researchers limited their investigation to the effect of positively and negatively chirped pulses. The results are presented in Fig. 14. In these ERG traces, one can recognize the characteristic A-wave (negative at early times) and B-wave (positive at later times) signals. The former is mainly attributed to cone activity and the latter to second-order retinal neurons from cones. These results unambiguously establish the possibility to efficiently acquire the visual response *in vivo* using pulsed ultra-short laser excitation, paving the way to experiments aiming at the control of vision based on phase-coherently prepared states of light.

### B. Rhodopsin: Femtosecond *cis-trans* isomerisation induces structural rearrangements on the millisecond timescale

**FIG. 15.**
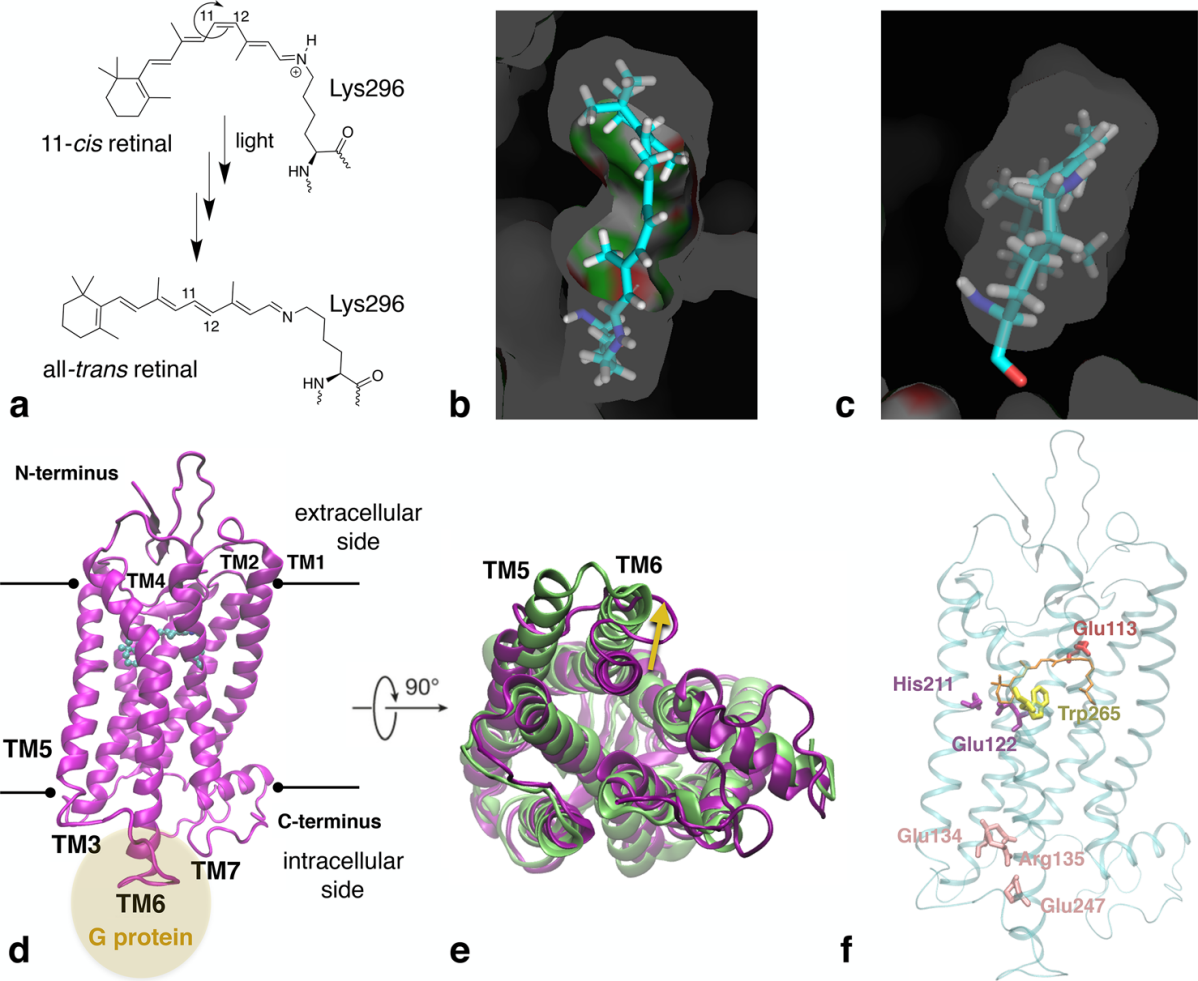
Rhodopsin's activation mechanism. (a) 11-*cis* to all-*trans* photoisomerisation and later deprotonation of covalently bound retinal in rhodopsin due to exposure to light. (b) View from the extracellular region of the retinal moiety in the active site. (c) View of the retinal moiety, turned 90° compared to image (b), with the Lys296 part located in front. (d) X-ray structure of inactive rhodopsin (PDB code 1U19). The black lines indicate the location of the cell membrane. The yellow circle shows the location of the G-protein interaction site. (e) Alignment of the active and inactive conformation of rhodopsin, viewed from the intracellular side. Corresponding PDB codes are listed in the same colour as the colour of the protein structure. The main structural changes due to activation are indicated by yellow arrows. (f) X-ray structure of inactive rhodopsin (PDB code 1U19) in which specific regions that are especially affected by retinal's *cis*-*trans* isomerisation are highlighted. The retinal moiety is shown in orange.

In the outer segment of rod cells in the retina of vertebrate eyes, the protein rhodopsin is present in high concentrations.^139^ In the dim light, the membrane-embedded rhodopsin is able to convert photons into a signal for the brain, which results in eyesight. Rhodopsin belongs to class A of the G-protein-coupled receptor (GPCR) family, which has a large impact on the function of the human body, as these membrane proteins enable communication between the intracellular and extracellular side of a cell.^140^ Class A G-protein-coupled receptors consist of seven trans-membrane helices, three intracellular and three extracellular loops, as well as a C-terminus and an N-terminal region [Fig. 15(d)]. Active GPCRs are able to stimulate or inhibit several proteins in the cytosol via interactions with G proteins in the intracellular region.^141^ For several decades, rhodopsin has been intensely studied as a prototype system to understand the activation process of GPCRs. Besides being viewed as an example protein for other class A GPCRs, rhodopsin also bears other properties of interest, such as low basal activity, a covalently bound chromophore in the active site, and a high yield of around 65% for the photoconversion of the chromophore's inactive 11–*cis*-retinal state to its active *trans* configuration [Fig. 15(a)].^142^

The *cis-trans* isomerisation of the chromophore initiated by photon exposure is the trigger for rhodopsin's conversion from its inactive state to its active metarhodopsin II conformation that interacts with G proteins in the cytosol. The ultrafast *cis-trans* photoisomerisation takes place on the femtosecond timescale in the extracellular part of the protein, while the structural rearrangements of rhodopsin's intracellular region that lead to the active state occur on the millisecond timescale (Fig. 15).^143–147^ Hence, eight orders of magnitude in time scales are involved to activate the entire protein due to the transduction of the external signal from the extracellular to the intracellular region. X-ray crystallography experiments have elucidated the structure of inactive (dark state) rhodopsin as well as several intermediates that lead to the fully active structure.^148–153^ However, not all intermediates of the activation pathway have been resolved and protonation reactions that take place during rhodopsin activation are difficult to understand from experimental data alone. Therefore, besides experimental studies, a significant number of computational studies have investigated the chromophore's configurational changes during protein activation as well as the impact of the structural change of the chromophore on the active site and the rest of the protein to obtain a better understanding of the chromophore's conversion from an inverse agonist to an agonist.^154–159^

Early studies of the photoisomerization reaction dynamics based on a restricted-open shell Kohn-Sham approach^160^ showed that the *cis-trans* isomerisation of the 11–*cis* retinal moiety that initiates rhodopsin activation is highly impacted by the shape of the non-polar active site. While in solution, *cis-trans* isomerisation can also occur around other bonds than the C11-C12 bond (Fig. 15),^161,162^ rhodopsin's tight active site [Figs. 15(b) and 15(c)] induces a pre-twist of the C10-C11-C12-C13 dihedral angle, which favours highly selective isomerisation around C11-C12.^160,163^ Within few hundreds of femtoseconds, the system relaxes from the first excited state via a conical intersection to the ground state. This process can be influenced and tuned by an external field.

To accomplish this, the local control theory (LCT)^164–166^ has been employed to obtain an optimal/anti-optimal pulse at each time step of propagation which maximizes/minimizes the *cis-trans* isomerization yield of retina. The underlying nuclear quantum wavepacket has been propagated with the multi-configuration time-dependent Hartree (MCTDH) method.^167–170^ A two-state two-mode vibronic coupling Hamiltonian, proposed by Hahn and Stock,^171,172^ has been employed to simulate ultrafast non-adiabatic dynamics of retinal in rhodopsin. The model includes the two energetically lowest electronic states and the two vibrational modes consisting of one tuning (torsional) mode, which brings in the 11–*cis* to all-*trans* transformation, and one coupling mode, which mainly involves C-C backbone stretching motion. Despite being derived from spectroscopic data, Hahn-Stock model has been shown, by a number of computational studies,^173–176^ to be quite successful in reproducing the main features of the underlying ultrafast dynamics.

**FIG. 16.**
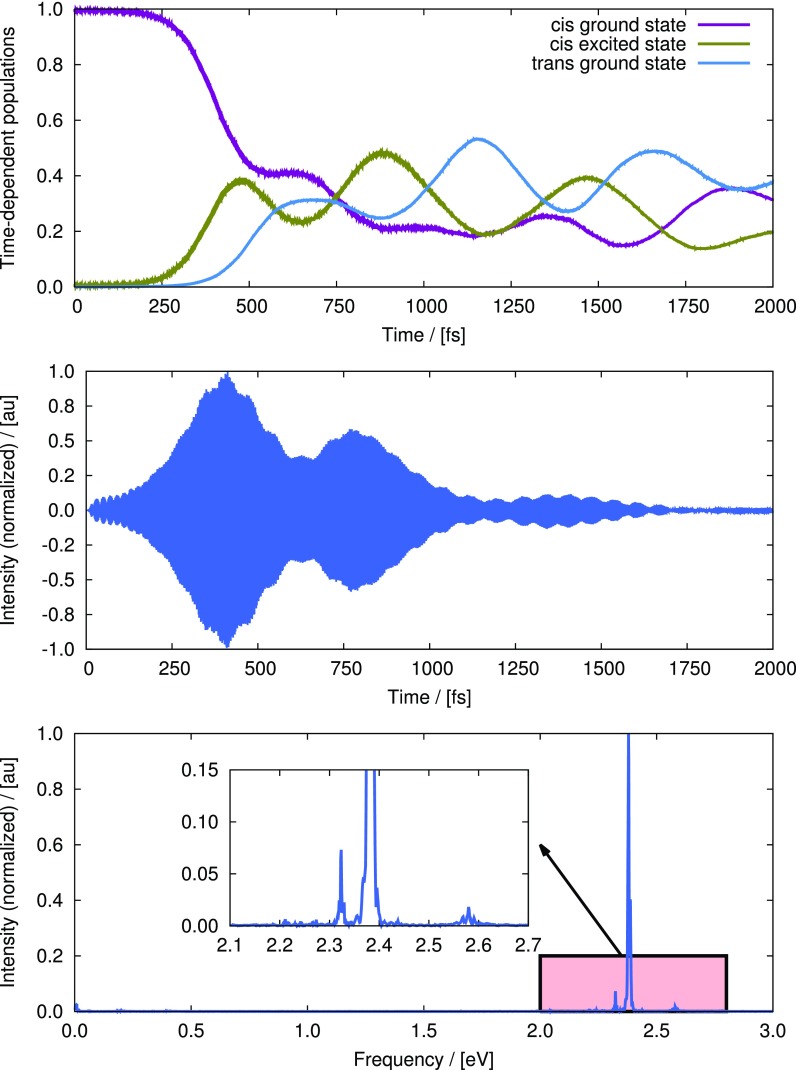
Results of an MCTDH/LCT simulation of retinal using Hahn-Stock model with an aim to maximize *trans* isomerized product. Top panel: time-dependent expectation values state-projection operators. The dark-magenta line corresponds to the ground electronic state in the *cis* configuration, the olive-green line corresponds to the first excited state in the *cis* configuration and the blue line corresponds to the first excited state in the *trans* configuration. Middle panel: the optimal pulse, in time domain, obtained for a propagation up to 2000 fs which maximizes the transfer of population to the *trans* ground state. Bottom panel: the Fourier transform of the time-domain pulse. Inset shows an enlarged view of an important part of the spectrum.

To maximize the *cis-trans* isomerization yield, the initial wavepacket has been prepared in the ground-electronic state equilibrium geometry corresponding to *cis* configurations. The results from a propagation of up to 2000 fs are shown in Fig. 16. The top panel of Fig. 16 shows the time-evolution of populations of the ground (magenta line) and the first excited electronic states (olive-green line) in the *cis* configuration and the same for the ground electronic state in the trans configuration (blue line). These quantities have been computed as the expectation values of the projection operators corresponding to the states of interest, where a *cis* configuration has been defined by the value of the torsional coordinate in the range [−π/2,π/2] and a *trans* configuration is defined by a range of [π/2,3π/2] of the same coordinate. The middle panel of Fig. 16 depicts the optimal pulse in time-domain which maximizes the quantum yield. The pulse gains appreciable intensity approximately after 200 fs and remains to be active essentially up to 1000 fs. The time-dependent populations (see the top panel) also change accordingly, while the dynamics of the wavepacket after 1000 fs is mainly controlled by the field-free time-dependent Schrödinger equation (TDSE). The bottom panel of Fig. 16 is the Fourier transform of the time-domain pulse. The highly intense peak close to 2.4 eV corresponds to the vertical excitation energy at the Franck-Condon point. A few low intensity frequencies also appear around the Rabi frequency; see the inset of the bottom panel of Fig. 16 for an enlarged view. These additional frequencies can be assigned to the excitation of the coupling mode in the excited electronic state and possibly with the existence of $\nu_{1}+n\nu_{2}$ type combination bands and overtones of it. Overall, ∼40% yield could be achieved at the end of the 200 fs propagation time. The rather low yield, compared with the experimental value, could be explained by the simplicity and the empirical nature of the model.

**FIG. 17.**
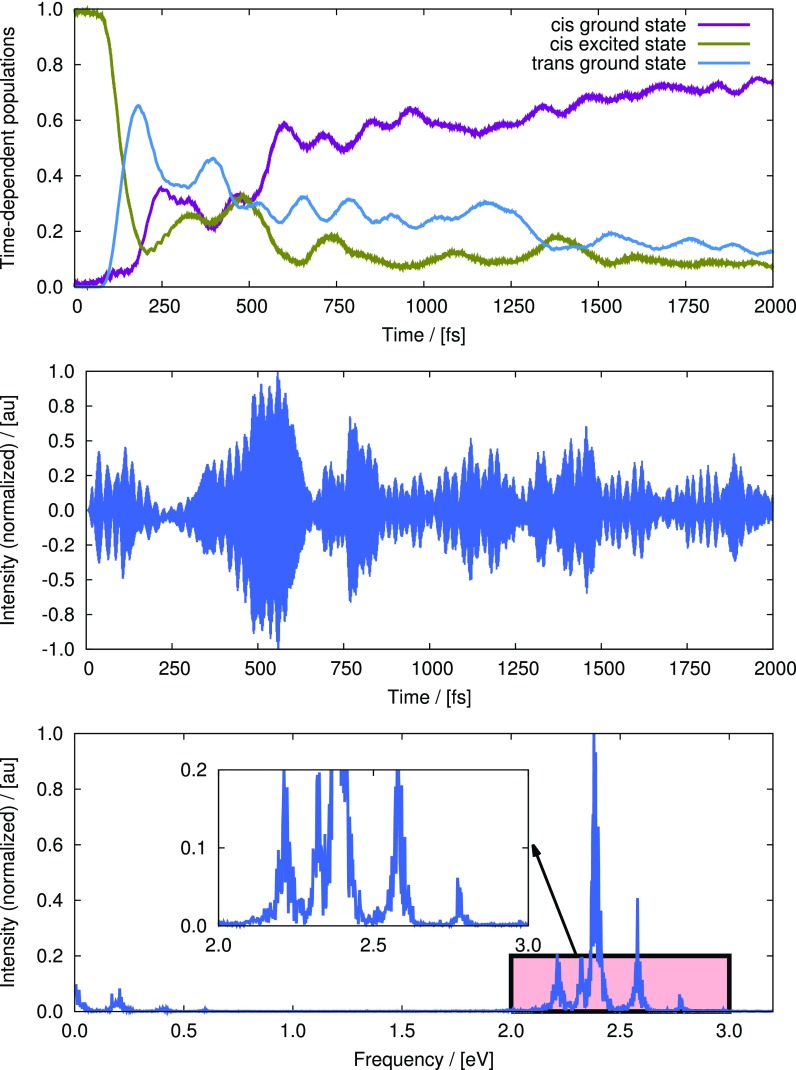
Results of an MCTDH/LCT simulation of retinal using Hahn-Stock model with an aim to minimize *trans* optimized product. Top panel: Time-dependent expectation values state-projection operators. The dark-magenta line corresponds to the ground electronic state in the *cis* configuration, the olive-green line corresponds to the first excited state in the *cis* configuration and the blue line corresponds to the first excited state in the *trans* configuration. Middle panel: The optimal pulse, in time domain, obtained for a propagation up to 2000 fs which maximizes the transfer of population to the *trans* ground state. Bottom panel: the Fourier transform of the time-domain pulse. Inset shows an enlarged view of an important part of the spectrum.

To further establish the ability to optically control the dynamics of the photoisomerization reaction, we also report the results of an anti-optimal MCTDH/LCT simulation. To this end, the initial wavepacket has been prepared in the first excited electronic state. To minimize *trans* product formation, an anti-optimal pulse has been computed which maximizes the transition from the first excited to the ground electronic state within the *cis* configurations. The top panel of Fig. 17 shows the time-dependent populations of different states. The simulation starts with a 100% population in the *cis* excited state (olive-green line). With time, the *trans* ground state (blue line) starts to obtain population and reaches its maximum at ∼200 fs. Following this event, some population transfer to the *cis* ground state (magenta line) and some back transfer to the cis excited state have been observed, which happen mainly due to the branching of the wavepacket by the conical intersection. The pulse (middle panel of Fig. 17) becomes effective from ∼300 fs. Thereafter, a steady increase of the population of *cis* ground state is observed, which continues until the end of the simulation (2000 fs). At the end of the simulation, the population of the *trans* ground state decreases to almost 10%. The underlying dynamics of this minimization can be described by the fact that once the wavepacket reaches the *cis* ground state (by the action of the pulse), it cannot escape the well due to the large potential-energy barrier between the *cis* and the *trans* minima in the ground electronic state. This also explains the steady increase in the *cis* ground state population after ∼400 fs. The bottom panel of Fig. 17 shows the Fourier transform of the time-domain pulse. Apart from the highly intense Rabi frequency, appearing almost at the same position as for the optimal simulation (bottom panel of Fig. 16), we also see a few other frequencies of considerable intensities. It is difficult to assign all the frequencies unambiguously. On the one hand, some of them may correspond to the decreasing vertical energy gaps that the wavepacket encounters as it proceeds to the conical intersection. On the other hand, some of the peaks may correspond to the excitation of the coupling mode. Overall, it can be stated that with an optimized pulse, it is possible to achieve some control on the quantum yield of the photoisomerization dynamics of retina in rhodopsin.

The structural relaxation after photoisomerization can be followed via classical molecular dynamics simulations with force-matched force fields^177^ up to the microsecond time scale^158,163^ and in agreement with experimental observations, distinct intermediates can be detected. These simulations show that after photoisomerisation, a strained *all*-trans configuration of the chromophore, photorhodopsin, gradually alters its structure and orientation in the active site to a more relaxed conformation. The first short-lived intermediate after a few picoseconds is bathorhodopsin in which the chromophore has released strain, but still includes a highly distorted *all*-trans configuration. Bathorhodopsin can form an equilibrium with a blue-shifted intermediate (BSI) on the nanosecond time scale, which then leads to a more relaxed conformation called lumirhodopsin after a few hundred nanoseconds. The alterations in the chromophore's configuration and orientation during relaxation induce spectral shifts between the several short-lived intermediates that are sampled after *cis-trans* isomerisation.^178^ These computed vertical excitation spectra are in excellent agreement with experimental results. The molecular factors that are responsible for this spectral tuning have been of special interest and many different possibilities have been proposed during the years. By applying machine learning algorithms to a large comprehensive dataset generated via classical and mixed quantum mechanical/molecular mechanical (QM/MM) simulations, it was recently possible to identify a minimal set of molecular descriptors in an unbiased way. This study shows that the principal factors responsible for the spectral tuning are intramolecular structural features such as the bond length alternation but also few descriptors that capture the relative orientation of the chromophore with respect to the active site pocket [Fig. 15(a)].^179–181^

Further relaxation of the chromophore structure induces deprotonation of Lys296's protonated Schiff base (PSB) and protonation of the counter ion Glu113 [Figs. 15(a) and 15(e)].^182^ A recent computational study shows that besides Glu113 and the chromophore, also active site water molecules as well as Gly90 and Thr94 play significant roles during deprotonation of the chromophore.^181^ These results emphasise the importance of the residues in the active site as well as the involvement of the environment on the activation pathway of rhodopsin.

The PSB deprotonation initiates further straightening of the *all*-trans retinal configuration, which induces helix displacement as well as a change in the position of Trp265^183^ and a rearrangement of the hydrogen-bond network around Glu122 and His211 [Fig. 15(e)].^184,185^ These signature changes that take place on the millisecond timescale transduce the structural rearrangements in the active site from the extracellular region to the core of the protein. On the intracellular side, the isomerisation ultimately affects the “closed” interaction site for G proteins that includes the conserved residues Glu134, Arg135, and Glu247, called the ionic lock [Fig. 15(e)]. As a part of the activation process, the salt bridges Glu134-Arg135 and Arg135-Glu247 are broken and Glu134 is protonated, leading to an “open” conformation of the intracellular region, the signalling state metarhodopsin II [Figs. 15(c)–15(e)].^149,152,186^ Rhodopsin's meta II configuration is able to interact with G proteins in the cytosol that will augment the external signal, as one active rhodopsin can stimulate multiple G proteins, which will ultimately lead to vision.

## V. SUMMARY AND OUTLOOK

The current review summarizes the dynamical studies on systems ranging from small single molecules in the gas phase to large proteins in solution over several orders of magnitude in time. Some of the systems (PDZ, Myoglobin, Insulin, and Rhodopsin) can be considered as pivotal for characterizing the intimate links between structure, dynamics, and energetics. In all of them, perturbations on short time scales (fs to ps) lead to ensuing functional dynamics on multiple longer time scales, spanning several orders of magnitude. For example, in the silver-coordinated pentapeptide (HG3W), electron transfer and subsequent proton transfer dynamics span 8 orders of magnitude. Coincidentally, a similar time scale is required in rhodopsin to activate the entire protein. This comparison shows that the time scale for “information transfer” does not necessarily scale with the (physical) size of the system. Rather, in HG3W, the long time dynamics is governed by the fact that after dissociation of the silver ligand, the peptide is trapped in a conformation, which is not suited for subsequent proton transfer. On the other hand, if the HG3W peptide is allowed to freely sample thermal conformations, structures competent for PT are accessed on much shorter time scales. Hence, the silver-bound HG3W structure is frustrated.

Similarly, reaction mechanisms, product channels, and reaction kinetics can change appreciably along seemingly small changes in a specific system. While H_2_SO_4_ and HSO_3_Cl readily eliminate H_2_O and HCl upon vibrational overtone excitation, respectively, HF-elimination from HSO_3_F is not observed at all. The reason is the different coupling between the internal degrees of freedom and differences in the time scale for energy redistribution upon vibrational excitation. This example highlights the fact that studying chemically related systems (here –F, –Cl, or –OH substitution; or amino acid mutation in proteins) using identical methods can lead to interesting and important insights.

It is also interesting to note that depending on the means by which energy is provided to a system, the same physical process can display different subsequent dynamics and kinetics. As an example, NO-rebinding to Mb has been studied using UV, optical, and infrared excitation to dissociate the ligand from the heme-iron. Depending on the excitation and probe wavelengths used, the number and magnitude of the rebinding timescales and the rebound fraction differ.^118^ This finding is probably related to the fact that coupling strengths between the excited degrees of freedom (e.g., electronic or infrared) and those responsible for driving the reaction (bond stretch and ligand translation/rotation) differ for the various excitation wavelengths. Given the finding that the type of perturbation applied to a system also imprints on its long-time dynamical response, it is evident that characterizing the short time dynamics is essential and mandatory for a functional understanding of such systems, which eventually should lead to modifying and influencing them in rational ways.

One notable development on the computational side is the use of more coarse grained approaches to describe the long-time dynamical evolution of complex systems. Such approaches are based on an ensemble of long-time (several hundred nanoseconds or longer) atomistic trajectories. The configurations sampled are first clustered using one of the many available clustering algorithms. This leads to a finite number of “basins.”^187–191^ Based on such a (nonunique) decomposition of state space, a Markov model can be constructed based on a transition network analysis.^192–195^ The kinetics between the nodes can be recovered by a Master equation^192^ or by kinetic Monte Carlo (KMC) methods.^196–200^ Such transition networks have found several applications in protein folding,^73,201–208^ enzyme catalysis,^209,210^ ligand migration,^211–214^ and studies of electron spin resonance.^215^

In most of the present applications, the focus was on a rate coefficient *k* for a particular process to occur. Within equilibrium theory, *k* is related to an activation free energy $\Delta G^{‡}=\Delta H^{‡}-T\Delta S^{‡}$. In many concrete applications (e.g., ligand design, protein engineering), one specific aim is to accelerate *k*, which is equivalent to reducing $\Delta G^{‡}$. Hence, understanding and characterizing the functional dynamics of a complex chemical or biological system at a molecular level over extended time scales should result in providing guidelines and insight on how to adapt the system's architecture to (a) better (i.e., more efficiently) execute its task or (b) carry out a different/related function.

While much has been learned about how to control the enthalpic part in a chemical system—i.e., the interaction strength between molecular building blocks, for example, through chemical modification—controlling entropy has turned out to be exceedingly difficult. Nature itself has solved this problem for particular classes of systems, for example, for proteins operating under extreme conditions (cold-adapted or hyperthermophilic proteins). Cold-adapted proteins invariably have a lower enthalpy and a more negative entropy of activation than their orthologs, which operate at ambient conditions. Hence, nature appears to have compensated the increased enthalpic energy by a more favourable entropic contribution. The different contributions provided by entropy and enthalpy depending on temperature imply a higher rate at low temperature for cold-adapted proteins which is physiologically desirable. The analysis of MD simulations^216^ suggests that the differences between cold-adapted proteins and their warm-adapted counterparts is in the flexibility of the outer parts of the protein (at the protein-water interface) rather than in the protein active site. In other words, protein surface rigidity/flexibility should be able to tune the enthalpic and entropic parameters contributing to $\Delta G^{‡}$.

Another example which links entropic factors to protein function is the very recent study^217^ of the chromosomal zinc-regulated repressor (CzrA) for which it was found that DNA binding leads to an increase in methyl side-chain flexibility and stabilizes the complex entropically. Nuclear magnetic resonance experiments of the axial order parameter ($S_{\text{axis}}^{2}$) for all methyl groups provide information about the motional freedom of the –CH_3_ groups on the pico- to nanosecond time scale. The $S_{\text{axis}}^{2}$ parameter is considered to be a measure for the conformational entropy and can therefore report on the loss or increase of order around particular regions in a protein. It was found that upon binding of Zn (the allosteric effector of CzrA) to the protein, most methyl groups are unaffected, whereas several show a change which sums up to a small net decrease in flexibility (−TΔS), indicative of a stiffening of the protein. Temperature dependent NMR-measurements found conditional side chain motions, which have their origin in the release of restrictions (i.e., higher entropy) imposed on rotamer populations by neighboring residues. The microsecond time scale dynamics (probed by ^13^C NMR-relaxation dispersion experiments) is compatible with a model in which Zn locks CzrA in a conformational substate, i.e., a mechanism based on conformational selection. Overall, the Zn-induced allosteric inhibition of DNA-binding to CzrA is driven by perturbations in a network of coupled equilibrium dynamics on the pico- to nanosecond time scale that gives rise to conformational interconversion dynamics on the *μ*s accompanied by a change in conformational entropy. Thus, the interplay between the fast internal dynamics (ps to ns) and sampling of different conformations (*μ*s to ms) could be associated with changes in the conditional motion induced by Zn, and thus may be a primary driver of allostery in CzrA.

The interplay between entropy and enthalpy is a long-standing, open question: how does entropy/enthalpy compensation manifest itself, what are the physical origins, and does it exist at all?^218,219^ Thermodynamically, the enthalpic component measures the change in heat released (or absorbed) upon changing from state *A* to state *B* (e.g., conformation A to conformation B or ligand-unbound to ligand-bound), whereas the entropic part quantifies the change in order. Entropy/enthalpy compensation therefore refers to the notion that a change in the enthalpic component is offset by a similar change in entropy with a net near-zero effect on the change in free energy ΔG. As an extreme example, complete compensation would imply that improving the binding strength of a ligand by introducing a favourable H-bonding interaction would be compensated by an unfavourable entropic contribution. Conversely, reducing the flexibility of part of a ligand (rotatable bond) would in turn lead to an energetic penalty. If entropy/enthalpy-compensation was a *general* feature of complex systems, tasks such as ligand-optimization (in drug discovery) or protein-engineering would be exceedingly difficult. In reality, such types of optimizations *are* difficult but not impossible as illustrated by the example of cold- or hot-adapted proteins.

For a comprehensive understanding of how structure, flexibility, energetics, and dynamics contribute to controlling ΔH and TΔS, close collaborations between experiment and computation of suitable paradigmatic systems covering time scales from femtoseconds out to biologically relevant time scales (seconds) are required. Such an endeavor requires experiments in multiple wavelength regions (X-ray, UV, IR, THz, and NMR) to unravel the complex interplay between energy, motion, and function. Using state-of-the art technology at all levels, such characterizations deems to be possible.
