# Publisher's Note: “Implications of short time scale dynamics on long time processes” (Struct. Dyn. 4, 061507 (2017)]

**DOI:** 10.1063/1.5020803

**Published:** 2018-01-12

**Authors:** Krystel El Hage, Sebastian Brickel, Sylvain Hermelin, Geoffrey Gaulier, Cédric Schmidt, Luigi Bonacina, Siri C. van Keulen, Swarnendu Bhattacharyya, Majed Chergui, Peter Hamm, Ursula Rothlisberger, Jean-Pierre Wolf, Markus Meuwly

**Affiliations:** 1 Department of Chemistry, University of Basel, Klingelbergstrasse 80, 4056 Basel, Switzerland; 2 Department of Applied Physics (GAP), University of Geneva, 22 Ch. de Pinchat, 1211 Geneva 4, Switzerland; 3 Institute of Chemical Sciences and Engineering, EPFL, Lausanne, Switzerland; 4 Department of Chemistry, University of Zurich, Zurich, Switzerland

This article was originally published online on 22 December 2017 without proper credit in the caption to Fig. 15. The online version of the article was corrected on 28 December 2017. The corrected figure caption appears below:

FIG. 15. Rhodopsin's activation mechanism. (a) 11-cis to all-trans photoisomerisation and later deprotonation of covalently bound retinal in rhodopsin due to exposure to light. (b) View from the extracellular region of the retinal moiety in the active site. (c) View of the retinal moiety, turned 90° compared to image (b), with the Lys296 part located in front. [(b) and (c) Reprinted with permission from S. C. Van Keulen, A. Solano, and U. Rothlisberger, J. Chem. Theory Comput. **13**(9), 4524–4534 (2017). Copyright 2017 American Chemical Society.] (d) X-ray structure of inactive rhodopsin (PDB code 1U19). The black lines indicate the location of the cell membrane. The yellow circle shows the location of the G-protein interaction site. (e) Alignment of the active and inactive conformation of rhodopsin, viewed from the intracellular side. Corresponding PDB codes are listed in the same colour as the colour of the protein structure. The main structural changes due to activation are indicated by yellow arrows. (f) X-ray structure of inactive rhodopsin (PDB code 1U19) in which specific regions that are especially affected by retinal's cis-trans isomerization are highlighted. The retinal moiety is shown in orange.

